# Genetic and phenotypic spectrum associated with IFIH1 gain-of-function

**DOI:** 10.1002/humu.23975

**Published:** 2020-01-14

**Authors:** Gillian I. Rice, Sehoon Park, Francesco Gavazzi, Laura A. Adang, Loveline A. Ayuk, Lien Van Eyck, Luis Seabra, Christophe Barrea, Roberta Battini, Alexandre Belot, Stefan Berg, Thierry Billette de Villemeur, Annette E. Bley, Lubov Blumkin, Odile Boespflug-Tanguy, Tracy A. Briggs, Elise Brimble, Russell C. Dale, Niklas Darin, François-Guillaume Debray, Valentina De Giorgis, Jonas Denecke, Diane Doummar, Gunilla Drake af Hagelsrum, Despina Eleftheriou, Margherita Estienne, Elisa Fazzi, François Feillet, Jessica Galli, Nicholas Hartog, Julie Harvengt, Bénédicte Heron, Delphine Heron, Diedre A. Kelly, Dorit Lev, Virginie Levrat, John H. Livingston, Itxaso Marti, Cyril Mignot, Fanny Mochel, Marie-Christine Nougues, Ilena Oppermann, Belén Pérez-Dueñas, Bernt Popp, Mathieu P. Rodero, Diana Rodriguez, Veronica Saletti, Cia Sharpe, Davide Tonduti, Gayatri Vadlamani, Keith Van Haren, Miguel Tomas Vila, Julie Vogt, Evangeline Wassmer, Arnaud Wiedemann, Callum J. Wilson, Ayelet Zerem, Christiane Zweier, Sameer M. Zuberi, Simona Orcesi, Adeline L. Vanderver, Sun Hur, Yanick J. Crow

**Affiliations:** 1 Division of Evolution and Genomic Sciences, Faculty of Biology, Medicine and Health, School of Biological Sciences, Manchester Academic Health Science Centre, University of Manchester, Manchester, United Kingdom; 2 Department of Biological Chemistry and Molecular Pharmacology, Harvard Medical School, Boston, Massachusetts; 3 Program in Cellular and Molecular Medicine, Boston Children’s Hospital, Boston, Massachusetts; 4 Division of Neurology, Children’s Hospital of Philadelphia, Philadelphia, Pennsylvania; 5 Paediatric Department, Dumfries and Galloway Royal Infirmary, Cargenbridge, United Kingdom; 6 Laboratory of Neurogenetics and Neuroinflammation, Institut Imagine, Paris, France; 7 Department of Neuropaediatrics, CHU & University of Liège, Liege, Belgium; 8 Department Clinical and Experimental Medicine, University of Pisa, Pisa, Italy; 9 IRCCS Fondazione Stella Maris, Pisa, Italy; 10 Université de Lyon, INSERM U1111, CIRI, Lyon, France; 11 Centre International de Recherche en Infectiologie, CIRI, Inserm, U1111, École Normale Supérieure de Lyon, Université Claude Bernard Lyon 1, Université de Lyon, Lyon, France; 12 Pediatric Immunology and Rheumatology, The Queen Silvia Children’s Hospital, Goteborg, Sweden; 13 Neuropédiatrie, Centre de référence Neurogénétique, Hôpital Trousseau, Sorbonne Université, Paris, France; 14 University Children’s Hospital, University Medical Center Hamburg Eppendorf, Hamburg, Germany; 15 Pediatric Neurology Unit, Metabolic Neurogenetic Service, Wolfson Medical Center, Holon, Israel; 16 Sackler Faculty of Medicine, Tel-Aviv University, Tel-Aviv, Israel; 17 Génétique Médicale, Université Paris Diderot, Paris, France; 18 Service de Neuropédiatrie et des Maladies Métaboliques, Centre de Référence Maladies Rares “Leucodystrophies”, Hopital Robert Debré, Paris, France; 19 Manchester Centre for Genomic Medicine, St Mary’s Hospital, Manchester University NHS Foundation Trust, Manchester, United Kingdom; 20 Department of Neurology, Stanford University School of Medicine, Stanford, California; 21 Faculty of Medicine and Health, Kids Neuroscience Centre, Brain and Mind Centre, Children’s Hospital at Westmead, University of Sydney, Sydney, Australia; 22 Department of Pediatrics, Institute of Clinical Sciences, Sahlgrenska University Hospital, University of Gothenburg, Gothenburg, Sweden; 23 The Queen Silvia Children’s Hospital, Sahlgrenska University Hospital, Gothenburg, Sweden; 24 Metabolic Unit, Department of Medical Genetics, CHU & University of Liège, Gembloux, Belgium; 25 Child Neurology and Psychiatry Unit, IRCCS Mondino Foundation, Pavia, Italy; 26 GHUEP, département de neuropédiatrie, Centre de référence neurogénétique mouvement anormaux de l'enfant, Hôpital Armand Trousseau, Paris, France; 27 Pediatric Neurology, The Queen Silvia Children’s Hospital, Goteborg, Sweden; 28 Paediatric Rheumatology, ARUK Centre for Adolescent Rheumatology, Institute of Child Health, University College London (UCL) Great Ormond Street Hospital, London, United Kingdom; 29 U.O. Neuropsichiatria Infantile, Fondazione IRCCS, Istituto Neurologico Carlo Besta, Milan, Italy; 30 Unit of Child Neurology and Psichiatry, ASST Spedali Civili of Brescia, Brescia, Italy; 31 Department of Experimental and Clinical Sciences, University of Brescia, Brescia, Italy; 32 Service de Médecine Infantile, Centre de Référence des maladies métaboliques de Nancy, CHU Brabois Enfants, Unité INSERM NGERE U1256, Nancy, France; 33 Department of Allergy/Immunology, Spectrum Health Helen Devos Children’s Hospital, Michigan State University College of Human Medicine, East Lansing, Michigan; 34 Department of Medical Genetics, CHU & University of Liège, Gembloux, Belgium; 35 Service de Neuropédiatrie, Centre Référence des Maladies Lysosomales, Hôpital Trousseau, Paris, France; 36 UF Génétique Médicale et Centre de Référence “Déficiences Intellectuelles”, Groupe Hospitalier Pitié-Salpêtrière, Paris, France; 37 The Liver Unit, Birmingham Women’s and Children’s Hospital NHS Foundation Trust, Birmingham, United Kingdom; 38 Metabolic Neurogenetic Service, Wolfson Medical Center, The Rina Mor Institute of Medical Genetics, Holon, Israel; 39 Service de pédiatrie, Centre Hospitalier Annecy Genevois, Pringy, France; 40 Department of Paediatric Neurology, Leeds General Infirmary, Leeds, United Kingdom; 41 Pediatric Neurology, Hospital Universitario Donostia, Universidad del País Vasco UPV-EHU, San Sebastian, Spain; 42 Departement de Génétique & Centre de Référence Déficience Intellectuelle de cause rare, GH Pitié-Sapêtrière, Paris, France; 43 Institut du Cerveau et de la Moelle épinière, INSERM U 1127, Sorbonne Universités, Paris, France; 44 Service de Neuropédiatrie, GHUEP, Hôpital Armand Trousseau, APHP, Paris, France; 45 Pediatric Neurology Research Group, Hospital Vall d'Hebron—Research Institute (VHIR), Universitat Autonoma de Barcelona, Barcelona, Spain; 46 Institute of Human Genetics, Friedrich-Alexander-Universität Erlangen-Nürnberg (FAU), Erlangen, Germany; 47 GRC n°19, pathologies Congénitales du Cervelet-LeucoDystrophies, CRMR maladies neurogénétiques, Sorbonne Université, Paris, France; 48 Service de Neuropédiatrie, Hôpital Trousseau, Groupe Hospitalier HUEP, Inserm U1141, Paris, France; 49 Developmental Neurology Unit, Fondazione IRCCS Istituto Neurologico Carlo Besta, Milan, Italy; 50 Paediatric Neurology, Starship Children’s Hospital, Auckland, New Zealand; 51 Pediatric Neurology Unit, V. Buzzi Children’s Hospital, Milan, Italy; 52 Neuropediatría, Hospital Universitari i Pôlitecnic La Fe, Valencia, Spain; 53 West Midlands Regional Clinical Genetics Service and Birmingham Health Partners, Birmingham Women’s and Children’s Hospitals NHS Foundation Trust, Birmingham, United Kingdom; 54 Department of Paediatric Neurology, Birmingham Women’s and Children’s Hospitals NHS Foundation Trust, Birmingham, United Kingdom; 55 National Metabolic Service, Starship Children’s Hospital, Auckland, New Zealand; 56 Paediatric Neurosciences Research Group, Royal Hospital for Children, Glasgow, United Kingdom; 57 School of Medicine, University of Glasgow, Glasgow, United Kingdom; 58 Department of Brain and Behavioural Sciences, University of Pavia, Pavia, Italy; 59 Sorbonne-Paris-Cité, Institut Imagine, Paris Descartes University, Paris, France; 60 Centre for Genomic and Experimental Medicine, MRC Institute of Genetics and Molecular Medicine, University of Edinburgh, Edinburgh, United Kingdom

**Keywords:** Aicardi–Goutières syndrome, IFIH1, MDA5, Singleton Merten syndrome, Type I interferonopathy

## Abstract

IFIH1 gain-of-function has been reported as a cause of a type I interferonopathy encompassing a spectrum of autoinflammatory phenotypes including Aicardi–Goutières syndrome and Singleton Merten syndrome. Ascertaining patients through a European and North American collaboration, we set out to describe the molecular, clinical and interferon status of a cohort of individuals with pathogenic heterozygous mutations in *IFIH1*. We identified 74 individuals from 51 families segregating a total of 27 likely pathogenic mutations in *IFIH1*. Ten adult individuals, 13.5% of all mutation carriers, were clinically asymptomatic (with seven of these aged over 50 years). All mutations were associated with enhanced type I interferon signaling, including six variants (22%) which were predicted as benign according to multiple in silico pathogenicity programs. The identified mutations cluster close to the ATP binding region of the protein. These data confirm variable expression and nonpenetrance as important characteristics of the *IFIH1* genotype, a consistent association with enhanced type I interferon signaling, and a common mutational mechanism involving increased RNA binding affinity or decreased efficiency of ATP hydrolysis and filament disassembly rate.

## 1 ∣ INTRODUCTION

In 2014, heterozygous gain-of-function mutations in *IFIH1* were reported to cause a spectrum of neuroimmune phenotypes including classical Aicardi–Goutières syndrome (AGS; Oda et al., 2014; Rice et al., 2014). *IFIH1* encodes interferon-induced helicase C domain-containing protein 1 (IFHI1; also known as melanoma differentiation associated gene 5 protein: MDA5) which senses viral double-stranded (ds) RNA in the cytosol, leading to the induction of a type I interferon-mediated antiviral response. Consequent to Mendelian determined gain-of-function, it is suggested that IFIH1 inappropriately senses self-derived nucleic acid as viral, leading to an autoinflammatory state classified as a type I interferonopathy (Ahmad et al., 2018; Crow & Manel, 2015). In 2015, a p.Arg822Gln substitution in IFIH1 was shown to cause Singleton Merten syndrome (SMS), an autosomal dominant trait variably characterized by a deforming arthropathy, abnormal tooth development and cardiac valve calcification, again in association with enhanced type I interferon signaling (Rutsch et al., 2015). Although it was initially considered that SMS was a distinct, mutation-specific disorder, subsequent reports indicate that SMS and the neuroinflammatory phenotypes seen in the context of IFIH1 gain-of-function constitute part of the same disease spectrum (Buers, Rice, Crow, & Rutsch, 2017; Bursztejn et al., 2015).

Type I interferonopathy associated *IFIH1* mutations are either absent from control databases, or only present at very low frequency. However, we have noted previously that in silico algorithms are not always reliable in differentiating *IFIH1* disease-causing variants from benign polymorphisms (Ruaud et al., 2018). Such difficulty in assigning molecular pathogenicity is compounded by marked variability in disease expression, sometimes even within the same family, and the observation of complete non-penetrance in certain pedigrees (Rice et al., 2014). Given this background, we considered it important to provide an update of our experience of sequencing individuals for pathogenic *IFIH1* mutations associated with a type I interferonopathy state. In total, we describe molecular and clinical data relating to 74 individuals from 51 families, identifying 27 likely pathogenic mutations that cluster close to the ATP binding region of the protein. Our data confirm variable expression and nonpenetrance as important characteristics of these mutant genotypes, and the consistent association with enhanced type I interferon signaling as assessed by interferon-stimulated gene (ISG) expression, referred to as the interferon score.

## 2 ∣ MATERIALS AND METHODS

### 2.1 ∣ Subjects

Patients were ascertained through direct contact and/or collaborating physicians across clinical research laboratories in the UK and France (Crow), the USA (Vanderver), and Italy (Orcesi). The study was approved by the Leeds (East) Research Ethics Committee (10/H1307/132), the Comite de Protection des Personnes (ID-RCB/EUDRACT: 2014-A01017-40), IRB study protocol (Myelin Disorders Bioregistry Project: IRB# 14-011236) and the local ethics committee of the IRCCS Mondino Foundation, Pavia, Italy (3549/2009 of 30/9/2009 and 11/12/2009; n.20170035275 of 23/10/2017). Amino acid substitutions were considered as pathogenic mutations when they were seen in the context of a neuroimmune/autoinflammatory state (including AGS, a spastic-dystonic syndrome, nonsyndromic spastic paraparesis or SMS), and when two or more of the following applied: observation of the same variant in an unrelated family; de novo occurrence; documented increase in ISG expression; in vitro data consistent with IFIH1 gain-of-function.

### 2.2 ∣ Mutational analysis

Mutations were identified on a variety of next-generation sequencing platforms. Where Sanger sequencing was undertaken, primers were designed to amplify the coding exons of *IFH1*, with mutation annotation based on the reference cDNA sequence NM_022168.2. Variants were assessed using the in silico programs SIFT (http://sift.jcvi.org), Polyphen2 (http://genetics.bwh.harvard.edu/pph2/), and CADD (https://cadd.gs.washington.edu), summarized in VarCards (http://varcards.biols.ac.cn/). Population allele frequencies were obtained from the gnomAD database (http://gnomad.broadinstitute.org).

### 2.3 ∣ Protein modeling

Molecular graphics figures were generated with PyMOL (Schrödinger) using the PDB coordinates (4GL2).

### 2.4 ∣ Interferon score

Interferon scores were calculated on the basis of the expression of ISGs according to previously published protocols. In brief, this involved either a quantitative reverse transcription-polymerase chain reaction (qPCR) analysis using TaqMan probes (Crow laboratory: Rice et al., 2013), or testing on a Nanostring platform (Vanderver laboratory: Adang et al., 2018+). In the former, the relative abundance of *IFI27* (Hs01086370_m1), *IFI44L* (Hs00199115_m1), *IFIT1* (Hs00356631_g1), *ISG15* (Hs00192713_m1), *RSAD2* (Hs01057264_m1), and *SIGLEC1* (Hs00988063_m1) transcripts was normalized to the expression levels of *HPRT1* (Hs03929096_g1) and *18S* (Hs999999001_s1). The median fold change of the six genes, compared to the median of 29 previously collected healthy controls, was then used to create an interferon score for each individual, with an abnormal interferon score being defined as greater than +2 standard deviations above the mean of the control group that is 2.466. Alternatively, the copy number of mRNA transcripts of the six ISGs listed above, and four housekeeping genes (*ALAS1, HPRT1, TBP*, and *TUBB*), was quantified using a Nanostring nCounter™ Digital Analyzer. The raw copy number of mRNA transcripts of each ISG was standardized using the geometric mean of the four housekeeping genes for each individual, and the six-gene interferon signature for each individual calculated using the median of the Z scores, with the result considered positive if ≥1.96 (>98th centile; one tail analysis).

### 2.5 ∣ Interferon reporter assay

The pFLAG-CMV4 plasmid encoding *IFIH1* has been described elsewhere (Rice et al., 2014). Indicated mutations were introduced using Phusion HiFi DNA polymerase. HEK 293T cells (ATCC) were maintained in 48-well plates in DMEM (Cellgro) supplemented with 10% fetal bovine serum and 1% L-glutamine. At 80% confluence, cells were cotransfected with pFLAGCMV4 plasmids encoding wild-type or mutant *IFIH1* (5 ng, unless indicated otherwise), interferon β (*IFNb*) promoter-driven firefly luciferase reporter plasmid (100 ng), and a constitutively expressed Renilla luciferase reporter plasmid (pRL-TK, 10 ng), by using Lipofectamine 2000 (Life Technologies) according to the manufacturer’s protocol. The medium was changed 6 hr after transfection, and cells were subsequently incubated for 18 hr with or without stimulation with poly(I-C) (500 ng; InvivoGen) using Lipofectamine 2000. Cells were lysed with Passive Lysis Buffer (Promega), and *IFNb* promoter activity was measured using a Dual-Luciferase Reporter Assay (Promega) and a Synergy 2 plate reader (BioTek). Firefly luciferase activity was normalized to *Renilla* luciferase activity Each experiment was performed in triplicate and data are presented as mean ± standard mean of error. Statistical significance was determined by two-tailed, unpaired Student’s *t*-test with *, **, and *** indicating *p* values <.05, <.01, and <.001, respectively. Expression levels of individual constructs were tested by western blot analysis.

## 3 ∣ RESULTS

### 3.1 ∣ Molecular data

**FIGURE 1:**
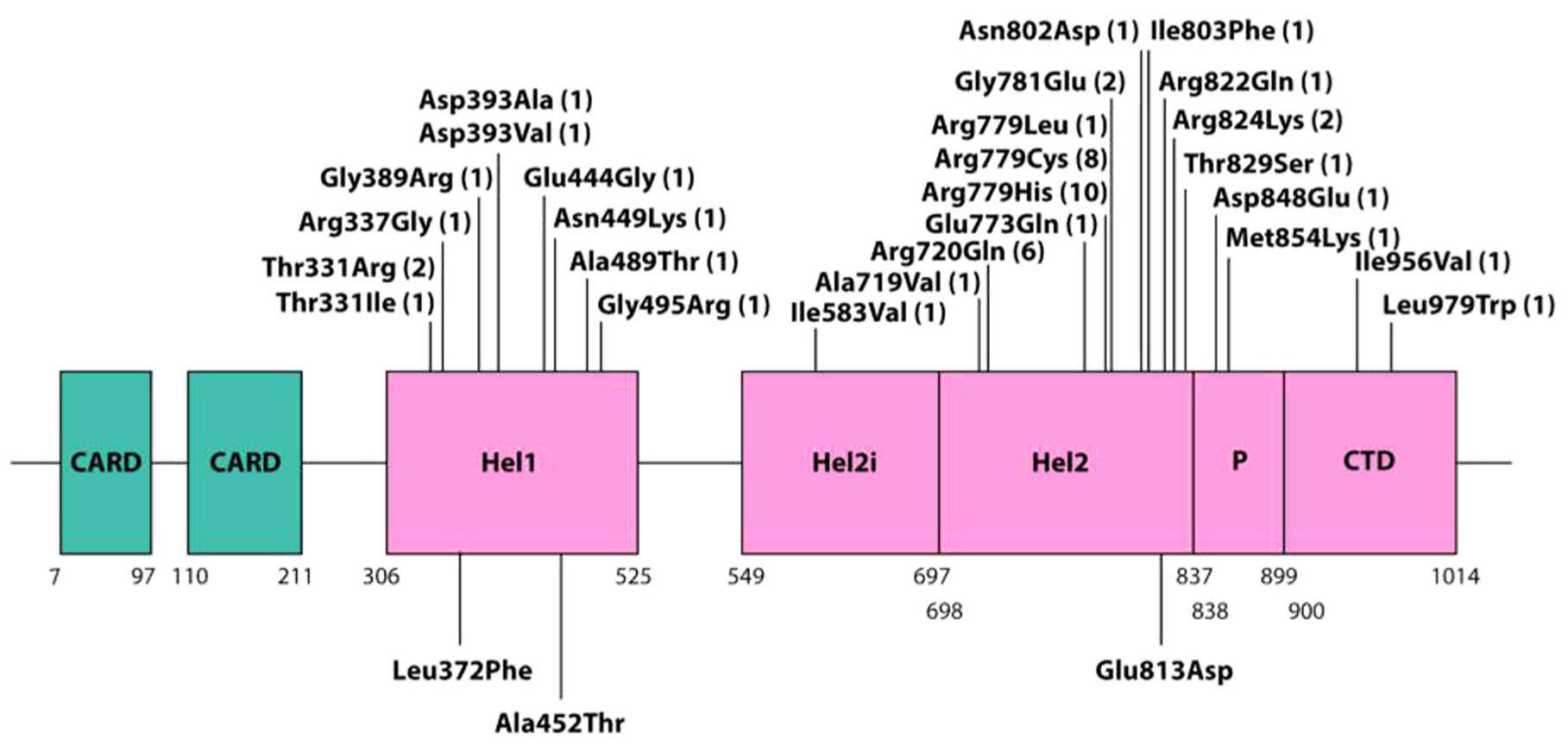
Schematic showing the positions of protein domains and their amino acid boundaries within the 1,025-residue IFIH1 protein. The 27 mutations ascertained in the present study are annotated, with the numbers in brackets indicating the number of families in which each mutation was observed. Three previously published mutations (p.Leu372Phe; p.Ala452Thr; p.Glu813Asp), not ascertained in our series, are also denoted (below the cartoon). CARD, caspase activation recruitment domain; Hel, helicase domain, where Hel1 and Hel2 are the two conserved core helicase domains and Hel2i is an insertion domain that is conserved in the RIG-I-like helicase family; P, pincer or bridge region connecting Hel2 to the C-terminal domain (CTD) involved in binding double stranded RNA

We collected data on 74 individuals from 51 families, identifying 27 distinct mutations in total (Figure 1; Table 1). Fourteen mutations were recorded in a single proband, seven in more than one individual belonging to a single-family, and six in more than one family. Of these six recurrent mutations, the p.Arg720Gln, p.Arg779Cys, and p.Arg779His substitutions were observed most frequently (6, 8, and 10 times, respectively). Twenty-two mutations were recorded to have occurred de novo in at least one individual, whilst four mutations were only ascertained in familial cases demonstrating autosomal dominant transmission (two mutations, p.Ala489Thr and p.Gly495Arg, were transmitted from a father in whom the mutation arose de novo). Three mutations, p.Thr331Arg, p.Arg779Cys, and p.Arg779His, was documented to have occurred both de novo, in association with severe, AGS-like, neurological disease, and in families with transmission across two or more generations.

For six putative mutations (p.Gly389Arg; p.Asn449Lys; p.Ile583Val; p.Ile803Phe; p.Asp848Glu; p.Ile956Val), in silico predictions using both SIFT and Poyphen2 suggested that the substitutions were benign, with relatively poor evolutionary conservation (Figure S1). However, all of these variants were novel (i.e., not recorded in gnomAD), and assays of interferon signaling (ISG expression and in vitro testing) indicate that they represent pathogenic mutations conferring gain-of-function (Table S1; Figure S2). Of note, four of these variants were seen in the context of a spastic paraparesis phenotype with no or minimal cognitive impairment. Clinical nonpenetrance was observed in three of these families (the other three variants arising in the proband de novo).

### 3.2 ∣ Clinical phenotype

**FIGURE 2:**
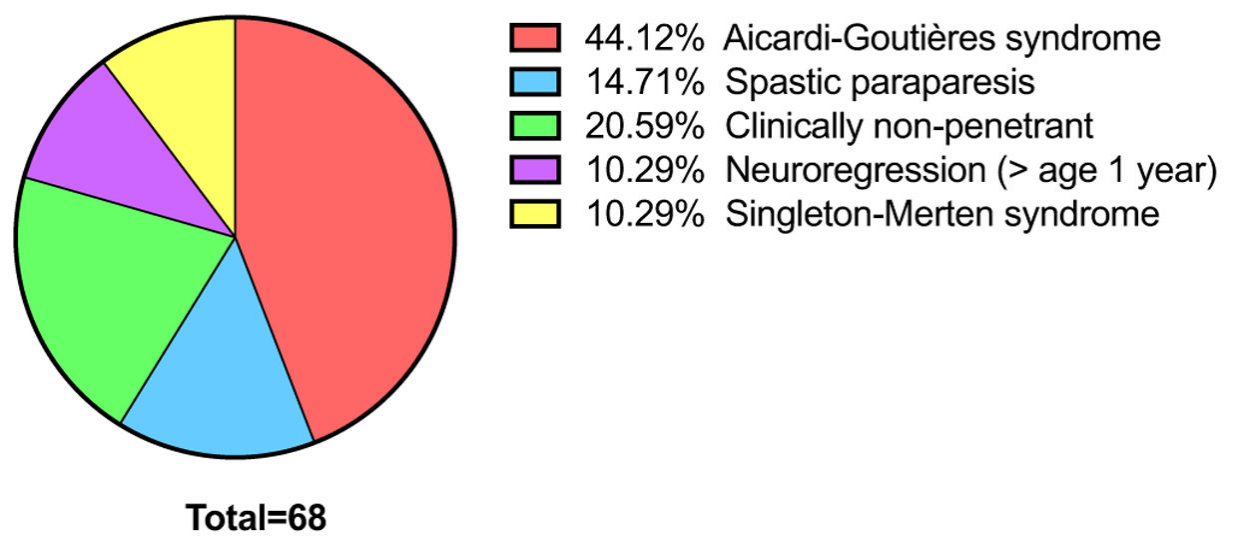
Overview of phenotypes observed in the *IFIH1*-mutation-positive cohort. Classification of 68 of 74 individuals according to phenotype. For clarity, six individuals displaying characteristics difficult to classify were omitted from this analysis

Consistent with previous data, we observed a spectrum of phenotypes in our cohort, encompassing classical AGS, less easily defined rapid neuroregression, a spastic-dystonic syndrome, spastic paraparesis, SMS, and clinical nonpenetrance (Figure 2; Table 2; Table S2). A single individual, AGS2222, experienced neonatal hepatitis and then developed chronic fibrotic liver disease in the absence of any other clinical features (note that this same variant was seen in another proband, AGS735, presenting with neuroregression at age 1 year). Unequivocal episodes of rapid neuroregression were noted in at least 20 patients, in seven of whom an acute loss of skills occurred after the age of 1 year on a background of completely normal development. Recognition/onset of symptoms was frequently later in patients with a spastic paraparesis phenotype, with one patient experiencing the development of lower limb spasticity beginning at 13 years of age (AGS531_P4). Six symptomatic patients were recorded to have died. Five of these individuals demonstrated a severe AGS phenotype with features obvious at, or soon after, birth that is indicating prenatal onset. One further deceased patient presented with neuroregression at age 15 months, and died suddenly of a cardiorespiratory arrest at 16 years of age, with pulmonary hypertension documented on postmortem examination. Ten individuals were reported as asymptomatic mutation carriers, across five mutations (p.Gly389Arg, p.Arg779Cys, p.Arg779His p.Asp848Glu, and p.Ile956Val), with seven aged over 50 years.

### 3.3 ∣ Interferon status

Where tested, all mutations (i.e., 26 of 27) were associated with increased expression of ISGs in peripheral blood (Table 1). Samples were unavailable for the single patient carrying the p.Glu773Gln substitution. This variant is not recorded in gnomAD, occurring de novo in the context of a phenotype compatible with IFIH1 upregulation, and conferring a gain-of-function in our in vitro assay (Figure S2). Considering all (51) mutation-positive individuals tested for ISG expression in the Crow laboratory (given that a direct comparison of results across laboratories is not possible), 109 of 117 values were positive (Table S3; Figure S3). Only one clinically symptomatic patient (AGS2154_1) demonstrated a negative interferon signature (on two of three occasions tested). The phenotype, in this case, was unusual; a child with white matter disease confined to the right cerebral hemisphere on MRI and no abnormal neurological signs on examination, having presented at age 8 years with headaches. We leave open the possibility that these two normal results, and three normal results from his mother, might be due to technical artifact, given that the samples had been stored for many months before testing. Sixteen samples from seven clinically nonpenetrant subjects exhibited an upregulation of interferon signaling, with two asymptomatic mutation carriers demonstrating normal interferon signatures (each tested on three occasions).

### 3.4 ∣ Modeling of IFIH1 gain-of-function mutations

**FIGURE 3:**
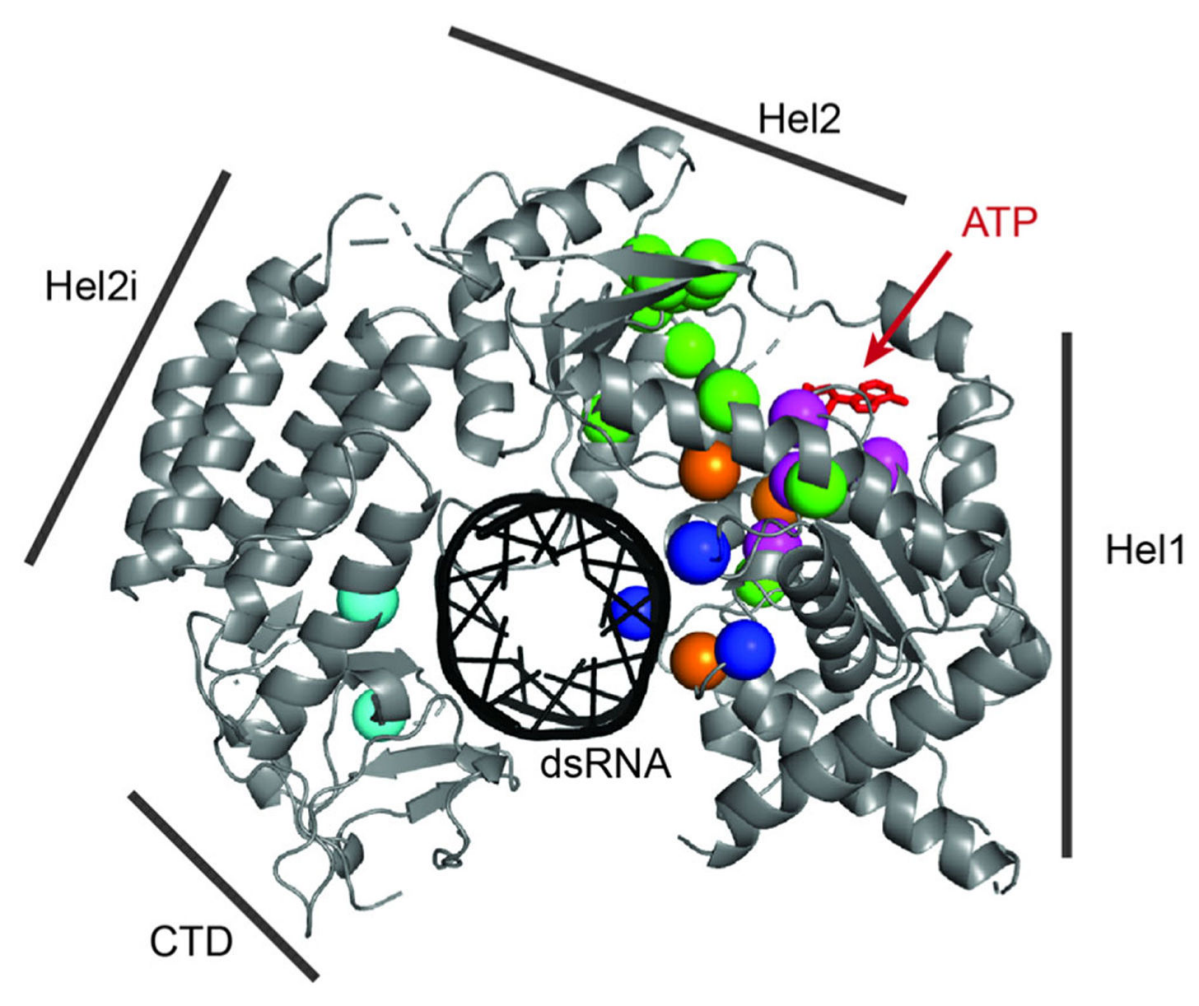
Mutation mapping. Structure of human IFIH1 (4GL2) in complex with double stranded RNA (dsRNA; blue stick model in the center). Only the RNA binding domain (helicase domain and C-terminal domain, CTD) are included in the crystal structure. Note that the helicase domain consists of Hel1, Hel2i, and Hel2. Mutations are indicated by spheres using the following color code: residues in the ATP binding pocket (magenta), residues in the dsRNA binding surface (blue), residues within the main cluster but not directly involved in RNA binding or ATP binding (green), residues outside the main cluster (cyan), and residues previously reported by others but not in our cohort (orange). We considered all 27 mutations reported here plus three previously published mutations (p.Leu372Phe; p.Ala452Thr; p.Glu813Asp) not ascertained in our series. Residues p.Arg822, p.Arg824, and p.Ile956 are not shown because they are disordered in the crystal structure, but are expected to be located in the ATP binding (p.Arg822 and p.Arg824) and RNA binding (p.Ile956) pockets

Modeling of the 27 mutations described here showed that most residues cluster near the ATP binding site within the helicase domain (Figure 3). Three mutations, p.Ileu583Val, p.Ileu956Val, and p.Leu979Trp were the only residues not situated in the cluster (colored cyan; only p.Ileu583Val and p.Leu979Val are shown since residue p.Ileu956 is disordered in the crystal structure). Within this main cluster, residues can be further categorized into three groups: those at the ATP binding pocket (magenta spheres), those in the double stranded RNA (dsRNA) binding surface (colored blue) and those not directly involved in either ATP or RNA binding (colored green). Three published mutations (p.Leu372phe; p.Ala452Thr; p.Glu813Asp; Table S4) not ascertained in our cohort are also located within the main cluster (colored orange), further supporting the importance of this region in the regulation of IFIH1 signaling activity.

## 4 ∣ DISCUSSION

Here we present data on 74 individuals, 41 previously unreported, from 51 families, with a putative gain-of-function mutation in IFIH1. Consistent with previous descriptions, we observed a spectrum of phenotypes, encompassing AGS, spastic-dystonia, spastic paraparesis, SMS and clinical nonpenetrance. Phenotypic variability was common, both in the context of familial inheritance and mutations seen recurrently across families so that no obvious genotype–phenotype correlations could be ascertained.

Acute regression was noted in almost one-third of symptomatic mutation carriers, occurring after the age of 1 year in seven patients demonstrating completely normal development to that time. Beyond acute regression, a slower onset of disease, and subsequent progression, was seen in patients demonstrating a spastic paraparesis phenotype. Together with the observation of clinical nonpenetrance (10:13.5% of 74 mutation-positive individuals in our series), with seven individuals identified to be apparently disease-free beyond the age of 50 years, these data suggest the importance of additive genetic factors and/or environmental triggers in determining phenotypic status. Although we did not formally record neuroimaging features in our cohort, white matter disease and intracranial calcification were observed frequently. Such imaging characteristics can be seen in the absence of overt neurological signs (see Bursztejn et al., 2015 and de Carvalho et al., 2017). Conversely, significant neurological disease, most typically spastic paraparesis, can occur in the context of normal brain and spinal imaging (e.g., the father in family AGS524).

Clinically manifest extraneurological illness was uncommon in our series, but there appears to be a real association between IFIH1 gain-of-function and lupus-like illness, autoimmune hepatitis, and hypothyroidism. Furthermore, psoriatic-like skin disease is a well-recognized feature of the SMS phenotype. As recently described (Adang et al., 2018), two patients included here were diagnosed with pulmonary hypertension, a feature which was not searched for in most patients and may be under-recognized.

We observed a strong association of mutation status with an enhanced expression of ISGs, with 109 of 117 samples from 51 patients being positive in the experience of one laboratory. A similar conclusion can be drawn from in vitro testing. As such, upregulated interferon signaling represents a reliable biomarker of IFIH1 gain-of-function, and can serve as an indicator of variant pathogenicity where doubt exists as to the significance of a molecular lesion. This is important given that we show here that in silico algorithms do not always accurately predict pathogenicity (involving 22% of the mutations that we recorded). Where tested, clinical nonpenetrance was also associated with a persistent upregulation of interferon signaling, with only two of nine such individuals nonpenetrant on ISG testing in blood. Whether these individuals demonstrate fluctuations in ISG expression is not known at this time.

Despite documented clinical nonpenetrance in some cases, all putative IFIH1 gain-of-function substitutions are rare, with only two of the 30 discrete mutations described here and in previous reports recorded in gnomAD. Furthermore, all ascertained type I interferonopathy associated mutations are missense variants, likely conferring increased sensitivity to a self-derived nucleic acid. Although premature termination mutations in the helicase domain are seen in control populations as common polymorphisms, none has been associated with a type I interferonopathy phenotype, further supporting the role of nucleic acid binding by the helicase domain in disease pathogenesis. Substitutions of the arginine residues at positions 720 and 779 were seen in six and 19 probands, respectively, in our series. Given the focus of our laboratories on pediatric neurological disease, our data are likely to subject to ascertainment bias. Indeed, although only observed once by us, the p.Arg822Gln mutation has been reported in an additional five pedigrees demonstrating a classical SMS phenotype (Pettersson et al., 2017; Rutsch et al., 2015).

IFIH1 is a member of the retinoic acid-inducible gene I (RIG-I) receptor family (del Toro Duany, Wu, & Hur, 2015). Recognition of cytoplasmic viral dsRNA by IFIH1 induces filament assembly along the dsRNA axis, with the helicase domains and C terminal domain responsible for RNA recognition. Filament formation then induces oligomerization of the tandem CARD domains (2CARD) of IFIH1, leading to the interaction with mitochondrial MAVS and subsequent induction of interferon and other proinflammatory cytokines. IFIH1 filament stability is intrinsically regulated by ATP hydrolysis, which is stimulated upon dsRNA binding. Mutations that impair ATP hydrolysis generally increase filament stability and, often, but not always, confer gain-of-function signaling activity. The clustering of mutations that we ascertained, and of a further three unique published mutations, near the ATP binding region likely highlights common mechanisms, perhaps increasing RNA binding affinity or decreasing the efficiency of ATP hydrolysis and the rate of filament disassembly.

Summarizing, IFIH1 gain-of-function is associated with a spectrum of phenotypes, occurring due to de novo mutations or transmitted as an autosomal dominant trait. Testing for an interferon signature in blood represents a useful biomarker in this context, which can aid in the interpretation of identified sequence variants.

## Supplementary Material

Supplements

## Figures and Tables

**TABLE 1:** Details of individual *IFIH1* mutations identified in the families included in the present data set

cDNA change	Protein change	Families (de novo inheritance; or, number of symptomatic and non- penetrant individuals where familial)	Associated phenotypes (‘/’ within family)(‘;’ between families)	Upregulation of interferon signalling	Assessment by interferon reporter assay	gnomAD	SIFT	Polyphen2	CADD score	Var- cards
c.992C>G	p.Thr331Arg	AGS674 (de novo); AGS1972 (2;0)	AGS-SMS; SMS	Yes	Yes (de Carvalho et al., 2017)	Novel	Deleterious 0	Probably damaging 1.000	29.7	22:23
c.992C>T	p.Thr331Ile	AGS1938 (3;0)	SMS	Yes	Yes (de Carvalho et al., 2017)	Novel	Deleterious 0	Probably damaging 1.000	31	22:23
c.1009A>G	p.Arg337Gly	AGS237 (de novo)	NR	Yes	Yes (Rice et al., 2014)	Novel	Tolerated 0.12	Probably damaging 1.000	26.8	17:23
c.1165G>A	p.Gly389Arg	AGS848 (2;1)	AGS/SP/CNP	Yes	Yes (this paper)	Novel	Tolerated 0.88	Benign 0.108	5.325	01:23
c.1178A>T	p.Asp393Val	AGS626 (de novo)	NR	Yes	Yes (Rice et al., 2014)	Novel	Deleterious 0.01	Probably damaging 0.998	28.6	16:23
c.1178A>C	p.Asp393Ala	AGS2586 (de novo)	AGS	Yes	No	Novel	Deleterious 0.03	Possibly damaging 0.913	24.8	12:23
c.1331A>G	p.Glu444Gly	AGS2669 (de novo)	AGS	Yes	Yes (this paper)	Novel	Deleterious 0	Probably damaging 1	31	23.23
c.1347C>G	p.Asn449Lys	AGS1001 (de novo)	SP	Yes	Yes (this paper)	Novel	Tolerated 0.64	Benign 0.163	13.91	03:23
c.1465G>A	p.Ala489Thr	AGS755 (3;0)^a^	CLL/AGS-SMS/SMS	Yes	Yes (Bursztejn et al., 2015)	Novel	Deleterious 0	Probably damaging 1.000	32	21:23
c.1483G>A	p.Gly495Arg	AGS524 (2;0)^a^	SP-LLD/SP	Yes	Yes (Rice et al., 2014)	Novel	Deleterious 0.01	Probably damaging 0.982	23.3	14:23
c.1747A>G	p.Ile583Val	AGS2369 (de novo)	AGS	Yes	Yes (this paper)	Novel	Tolerated 0.48	Benign 0.00	0.573	5.23
c.2156C>T	p.Ala719Val	Hm_1 (de novo)	AGS	Yes	No	Novel	Tolerated 0.07	Possibly damaging 0.949	27.1	09:23
c.2159G>A	p.Arg720Gln	AGS102 (de novo); AGS647 (de novo); AGS1504 (de novo); AGS2422 (NPDT); AGS2548 (de novo); LD_0982.0 (de novo)	AGS; SP	Yes	Yes (Rice et al., 2014)	Novel	Deleterious 0	Probably damaging 0.992	34	17:23
c.2317G>C	p.Glu773Gln	AGS2399 (de novo)	NR	NA	Yes (this paper)	Novel	Tolerated 0.27	Possibly damaging 0.743	24.8	13:23
c.2335C>T	p.Arg779Cys	AGS376 (NPDT); AGS723 (NPDT); AGS1004 (de novo); AGS1156 (de novo); AGS2154 (1;1); AGS2180 (de novo); AGS2507 (de novo); LD_1030.0 (de novo)	AGS-LLD; SP-ICC; NR; unilateral white matter disease/CNP; AGS	Yes	Yes (Rice et al., 2014)	Novel	Deleterious 0.01	Probably damaging 1.000	34	21:23
c.2336G>A	p.Arg779His	AGS163 (de novo); AGS259 (3;2); AGS1351 (de novo); AGS1509 (de novo); AGS2177 (1;2); Berg_1 (de novo); Orc_0098 (de novo); LD_1199.0 (de novo); LD_1381 (3;1); LD_1585.0 (de novo)	AGS; CNP; NR; SP	Yes	Yes (Rice et al., 2014)	1/244230	Tolerated 0.05	Probably damaging 0.994	28.9	19:23
c.2336G>T	p.Arg779Leu	LD_1067.0 (de novo)	AGS	Yes	No	Novel	Tolerated 0.06	Probably damaging 1.000	35	21:23
c.2342G>A	p.Gly781Glu	LD_0940.0 (de novo); LD_0943.0 (de novo)	NR; SP	Yes	No	Novel	Deleterious 0	Probably damaging 1.000	32	19:23
c.2404A>G	p.Asn802Asp	AGS2662 (de novo)	NR	Yes	No	Novel	Tolerated 0.22	Probably damaging 1.000	28.1	18:23
c.2407A>T	p.Ile803Phe	LD_1488.0 (de novo)	AGS	Yes	Yes (this paper)	Novel	Tolerated 0.24	Benign 0.043	11.8	04:23
c.2465G>A	p.Arg822Gln	AGS1514 (de novo)	SD-ICC	Yes	Yes (Rutsch et al., 2015)	6/244096	Deleterious 0	Probably damaging 1.000	35	23:23
c.2471G>A	p.Arg824Lys	AGS735 (de novo); AGS2222 (de novo)	NR; Isolated liver disease	Yes	No	Novel	Deleterious 0	Probably damaging 1.000	34	22:23
c.2486C>G	p.Thr829Ser	AGS1290 (2 siblings and NPDT)	AGS	Yes	No	Novel	Tolerated 0.73	Possibly damaging 0.512	16.61	12:23
c.2544T>G	p.Asp848Glu	AGS531 (3;2)	SP-ICC/CNP	Yes	Yes (Ruaud et al., 2018)	Novel	Tolerated 0.4	Benign 0.004	10.08	02:23
c.2561T>A	p.Met854Lys	AGS2081 (de novo)	AGS/SMS	Yes	No	Novel	Deleterious 0	Probably damaging 1.000	31	18:23
c.2866A>G	p.Ile956Val	AGS1430 (2;1)	SP-ICC/CNP	Yes	Yes (this paper)	Novel	Tolerated 0.77	Benign 0.004	3.576	06:23
c.2936T>G	p.Leu979Trp	LD_1346.0 (de novo)	AGS	Yes	Yes (this paper)	Novel	Deleterious 0.01	Probably damaging 1.000	26.6	16:23

*Note:* IFIH1 mutation annotation based on the reference complementary DNA sequence NM_022168.2.

Abbreviations: AGS, Aicardi–Goutières syndrome; CLL, Chilblain-like lesions; CNP, clinical nonpenetrance; ICC, Intracranial calcification; LLD, Lupus-like disease; NPDT, no parental DNA testing; NR, neuro-regression; SD, spastic dystonia; SP, spastic paraparesis; SMS, Singleton Merten syndrome.

a This mutation was shown to have been paternally inherited by the proband and to have occurred de novo in the proband’s father.

**TABLE 2:** Molecular and clinical data by family

Family	Individual	Sex	cDNA	Protein	Inheritance (number of mutation-positive individuals)	Previously reported (reference)	Clinical phenotype	Status at last contact (age in years)
AGS102	P1	M	c.2159G>A	p.Arg720Gln	De novo	Rice et al. (2014)	AGS	Deceased (2)
AGS163	P1	M	c.2336G>A	p.Arg779His	De novo	Rice et al. (2014)	AGS	Alive (13)
AGS237 (LD_0762)	P1	M	c.1009A>G	p.Arg337Gly	De novo	Rice et al. 2014; Adang et al., 2018	Neuroregression and SD starting at age 15 months	Deceased (16)
AGS259	P1	M	c.2336G>A	p.Arg779His	Familial (3)	Rice et al. (2014)	AGS	Alive (13)
	P2 (father of P1)	M					Clinically nonpenetrant	Alive (54)
	P3 (mother of P2)	F					Clinically nonpenetrant	Deceased (84)
AGS376	P1	M	c.2335C>T	p.Arg779Cys	No parental testing	Rice et al. (2014)	AGS with LLD	Deceased (3)
AGS524	P1	F	c.1483G>A	p.Gly495Arg	Familial (2)(shown to have occurred de novo in P2)	Rice et al. (2014); Hacohen et al. 2015; Crow et al. 2015; McLellan et al. 2018	SP with LLD and AQP4 + TM	Alive (10)
	P2 (father of P1)	M					Pure SP	Alive (39)
AGS531	P1	F	c.2544T>G	p.Asp848Glu	Familial (5)	Ruaud et al. (2018)	SP with ICC	Alive (13)
	P2 (brother of P1)	M					Clinically nonpenetrant	Alive (13)
	P3 (father of P1 and P2)	M					SP with ICC	Alive (40)
	P4 (brother of P3)	M					SP with ICC	Alive (38)
	P5 (father of P3 and P4)	M					Clinically non-penetrant	Alive (66)
AGS626	P1	M	c.1178A>T	p.Asp393Val	De novo	Rice et al. (2014)	Neuroregression and SD starting at 13 months	Alive (13)
AGS647	P1	M	c.2159G>A	p.Arg720Gln	De novo	Rice et al. (2014)	AGS	Alive (2)
AGS674	P1	M	c.992C>G	p.Thr331Arg	De novo	Unreported	SP-SMS overlap	Alive (14)
AGS723	P1	F	c.2335C>T	p.Arg779Cys	Mother negative; no paternal DNA	Unreported	SP with ICC	Alive (19)
AGS735	P1	M	c.2471G>A	p.Arg824Lys	De novo	Galli et al. 2018	Neuroregression and SD starting at 12 months	Alive (19)
AGS755	P1	M	c.1465G>A	p.Ala489Thr	Familial (3)	Bursztejn et al. (2015)	CLL	Alive (4)
	P2 (brother of P1)	M					AGS-SMS overlap	Alive (3)
	P3 (father of P1 and P2)	M					SMS-like	Alive (41)
AGS848	P1	M	c.1165G>A	p.Gly389Arg	Familial (3)	Unreported	AGS	Alive (8)
	P2 (father of P1)	M					SP	Alive (42)
	P3 (maternal grandmother of P2)	F					Clinically nonpenetrant	Alive (84)
AGS1001	P1	M	c.1347C>G	p.Asn449Lys	De novo	Unreported	SP	Alive (19)
AGS1004	P1	F	c.2335C>T	p.Arg779Cys	De novo	Unreported	AGS (neuroregression with onset at age 8 months)	Alive (8)
AGS1156	P1	M	c.2335C>T	p.Arg779Cys	De novo	Kothur et al. 2018	AGS (neuroregression with onset at age 8 months)	Alive (5)
AGS1290	P1	M	c.2486C>G	p.Thr829Ser	2 affected (no parental DNA)	Unreported	AGS	Alive (6)
	P2 (brother of P1)	M					AGS	Alive (4)
AGS1351	P1	F	c.2336G>A	p.Arg779His	De novo	Unreported	AGS	Deceased (2)
AGS1430	P1	M	c.2866A>G	p.Ile956Val	Familial (3)	Unreported	SP with ICC with onset at age 6 years	Alive (14)
	P2 (father of P1)	M					SP with onset at age 2 years	Alive (50)
	P3 (father of P2)	M					Clinically non-penetrant	Alive (72)
AGS1504 (LD_1175)	P1	F	c.2159G>A	p.Arg720Gln	De novo	Unreported	AGS	Alive (10)
AGS1509	P1	M	c.2336G>A	p.Arg779His	De novo	Unreported	AGS	Alive (8)
AGS1514	P1	M	c.2465G>A	p.Arg822Gln	De novo	Buers et al. (2017)	SD with ICC	Alive (6)
AGS1938	P1	F	c.992C>T	p.Thr331Ile	Familial (3)	de Carvalho et al. (2017)	SMS	Alive (18)
	P2 (mother of P1)	F					SMS	Alive (45)
	P3 (sister of P2)	F					SMS	Alive (27)
AGS1972	P1	F	c.992C>G	p.Thr331Arg	Familial (2)	de Carvalho et al. (2017)	SMS	Alive (9)
	P2 (father of P1)	M					SMS	Alive (47)
AGS2081	P1	M	c.2561T>A	p.Met854Lys	De novo	Unreported	SP-SMS overlap	Alive (12)
AGS2154 (LD_1240)	P1	M	c.2335C>T	p.Arg779Cys	Familial (2)	Unreported	Unilateral white matter disease with normal development	Alive (13)
	P2 (mother of P1)	F					Clinically nonpenetrant	Alive (40)
AGS2177	P1	M	c.2336G>A	p.Arg779His	Familial (3)		Neuroregression and SD starting at age 12 months	Alive (29)
	P2 (mother of P1)	F					Clinically nonpenetrant	Alive (62)
	P3 (sister of P1)	F					Clinically nonpenetrant	Alive (33)
AGS2180	P1	F	c.2335C>T	p.Arg779Cys	De novo	Unreported	AGS	Alive (4)
AGS2222	P1	M	c.2471G>A	p.Arg824Lys	De novo	Unreported	Isolated liver disease	Alive (9)
AGS2369	P1	M	c.1747A>G	p.Ile583Val	De novo	Unreported	AGS	Alive (10)
AGS2399	P1	M	c.2317G>C	p.Glu773Gln	De novo	Unreported	Neuroregression and SD starting at age 16 months	Alive (8)
AGS2422	P1	F	c.2159G>A	p.Arg720Gln	No parental testing	Unreported	SP	Alive (38)
AGS2507	P1	F	c.2335C>T	p.Arg779Cys	De novo	Unreported	AGS	Alive (1)
AGS2548	P1	M	c.2159G>A	p.Arg720Gln	De novo	Unreported	AGS	Alive (3)
AGS2586	P1	M	c.1178A>C	p.Asp393Ala	De novo	Unreported	AGS-like with frank regression at age 21 months	Alive (3)
AGS2662 (LD_1640)	P1	F	c.2404A>G	p.Asn802Asp	De novo	Unreported	Neuroregression and SD starting at age 11 months	Alive (1)
AGS2669	P1	M	c.1331A>G	p.Glu444Gly	De novo	Unreported	AGS	Deceased (0.5)
Hm_1	P1	F	c.2156C>T	p.Ala719Val	De novo	Unreported	AGS	Alive (2)
Berg_1	P1	F	c.2336G>A	p.Arg779His	De novo	Unreported	Neuroregression and SD starting at age 9 months	Alive (7)
Orc_0098	P1	M	c.2336G>A	p.Arg779His	De novo	Unreported	AGS	Alive (4)
LD_0940.0	P1	M	c.2342G>A	p.Gly781Glu	De novo	Unreported	Neuroregression and SD starting at age 15 months	Alive (5)
LD_0943.0	P1	F	c.2342G>A	p.Gly781Glu	De novo	Unreported	SP	Alive (14)
LD_0982.0	P1	M	c.2159G>A	p.Arg720Gln	De novo	Adang et al. (2018); Case 2	AGS	Alive (9)
LD_1030.0	P1	F	c.2335C>T	p.Arg779Cys	De novo	Unreported	AGS	Alive (5)
LD_1067.0	P1	M	c.2336G>T	p.Arg779Leu	De novo	Unreported	AGS	Alive (8)
LD_1199.0	P1	F	c.2336G>A	p.Arg779His	De novo	Unreported	AGS	Alive (4)
LD_1346.0	P1	M	c.2936T>G	p.Leu979Trp	De novo	Adang et al. (2018); Case 3	AGS	Deceased (0.4)
LD_1381 (Hart)	P1	F	c.2336G>A	p.Arg779His	Familial (4)	Unreported	SP	Alive (4)
	P2 (brother of P1)	M					SP	Alive (3)
	P3 (father of P1 and P2)	M					SP	Alive (32)
	P4 (father of P3)	M					Clinically nonpenetrant	Alive (68)
LD_1488.0	P1	F	c.2407A>T	p.Ile803Phe	De novo	Unreported	AGS	Alive (2)
LD_1585.0	P1	F	c.2336G>A	p.Arg779His	De novo	Unreported	AGS	Alive (5)

*Note:* IFIH1 mutation annotation based on the reference complementary DNA sequence NM_022168.2.

Abbreviations: AGS, Aicardi–Goutières syndrome; CLL, Chilblain-like lesions; F, Female; ICC, intracranial calcification; LLD, Lupus-like disease; M, Male; SD, spastic dystonia; SP, spastic paraparesis; SMS: Singleton Merten syndrome; TM, transverse myelitis
