# Supplementary material for: Genetic and phenotypic spectrum associated with IFIH1 gain-of-function
Source: Hum Mutat. Author manuscript; Available in PMC 2021 Apr 1. (PMC7457149; doi:10.1002/humu.23975)
Supplement: Supplements [file NIHMS1618607-supplement-Supplements.pdf]

Genetic and phenotypic spectrum associated with IFIH1 gain-of-function

Gillian I Rice,[1*] Sehoon Park,[2,3*] Francesco Gavazzi,[4] Laura A Adang,[4] Loveline A Ayuk,[5] Lien Van Eyck,[6] Luis Seabra,[6] Christophe Barrea,[7] Roberta Battini,[8,9] Alexandre Belot,[10,11] Stefan Berg,[12] Thierry Billette de Villemeur,[13] Annette E Bley,[14] Lubov Blumkin,[15,16] Odile Boespflug-Tanguy,[17,18] Tracy A Briggs,[1,19] Elise Brimble,[20] Russell C Dale,[21] Niklas Darin,[22,23] François-Guillaume Debray,[24] Valentina De Giorgis,[25] Jonas Denecke,[14] Diane Doummar,[26] Gunilla Drake af Hagelsrum,[27] Despina Eleftheriou,[28] Margherita Estienne,[29] Elisa Fazzi,[30,31] François Feillet,[32] Jessica Galli,[30,31] Nicholas Hartog,[33] Julie Harvengt,[34] Bénédicte Heron,[35] Delphine Heron,[36] Diedre A Kelly,[37] Dorit Lev,[16,38] Virginie Levrat,[39] John H Livingston,[40] Itxaso Marti,[41] Cyril Mignot,[42] Fanny Mochel,[43] Marie-Christine Nougues,[44] Ilena Oppermann,[14] Belén Pérez-Dueñas,[45] Bernt Popp,[46] Mathieu P Rodero,[6] Diana Rodriguez,[47,48] Veronica Saletti,[49] Cia Sharpe,[50] Davide Tonduti,[51] Gayatri Vadlamani,[40] Keith Van Haren,[20] Miguel Tomas Vila,[52] Julie Vogt,[53] Evangeline Wassmer,[54] Arnaud Wiedemann,[32] Callum J Wilson,[55] Ayelet Zerem,[15,16] Christiane Zweier,[46] Sameer M Zuberi,[56,57] Simona Orcesi,[25,58] Adeline L Vanderver,[4] Sun Hur,[2,3] Yanick J Crow,[6,59,60]

[1]Division of Evolution and Genomic Sciences, School of Biological Sciences, Faculty of Biology, Medicine and Health, University of Manchester, Manchester Academic Health Science Centre, Manchester, M13 9PT, United Kingdom

[2]Department of Biological Chemistry and Molecular Pharmacology, Harvard Medical School, Boston, MA 02115, USA

[3]Program in Cellular and Molecular Medicine, Boston Children's Hospital, MA 02115, USA

[4]Division of Neurology, Children's Hospital of Philadelphia, Philadelphia, PA, USA

[5]Paediatric Department, Dumfries and Galloway Royal Infirmary, Cargenbridge, United Kingdom

[6]Laboratory of Neurogenetics and Neuroinflammation, Institut Imagine, Paris, France

[7]Department of Neuropaediatrics, CHU & University of Liège, Belgium

[8]Dept. Clinical and Experimental Medicine, University of Pisa, Pisa, Italy
[9]IRCCS Fondazione Stella Maris, Pisa, Italy
[10]Université de Lyon, INSERM U1111, CIRI, Lyon, France
[11]Centre International de Recherche en Infectiologie, CIRI, Inserm, U1111, Université Claude Bernard Lyon 1, CNRS, UMR5308, École Normale Supérieure de Lyon, Université de Lyon, Lyon, France
[12]Pediatric Immunology and Rheumatology, The Queen Silvia Children's Hospital, Goteborg, Sweden
[13]Sorbonne Université, Neuropédiatrie, AP-HP, Hôpital Trousseau, Centre de référence Neurogénétique, Inserm U 1141, Paris, France
[14]University Children's Hospital, University Medical Center Hamburg Eppendorf, Hamburg, Germany
[15]Pediatric Neurology Unit, Metabolic Neurogenetic Service, Wolfson Medical Center, Holon, Israel
[16]Sackler Faculty of Medicine, Tel-Aviv University, Tel-Aviv, Israel
[17]Génétique Médicale, Université Paris Diderot, Paris, France
[18]Service de Neuropédiatrie et des Maladies Métaboliques, Centre de Référence Maladies Rares "Leucodystrophies", Hopital Robert Debré, Paris, France
[19]Manchester Centre for Genomic Medicine, St Mary's Hospital, Manchester University NHS Foundation Trust, Manchester, United Kingdom
[20]Department of Neurology, Stanford University School of Medicine, Stanford, California, USA
[21]Kids Neuroscience Centre, Brain and Mind Centre, Children's Hospital at Westmead, Faculty of Medicine and Health, University of Sydney, Australia
[22]Department of Pediatrics, Institute of Clinical Sciences, Sahlgrenska University Hospital, University of Gothenburg, Sweden
[23]The Queen Silvia Children's Hospital, Sahlgrenska University Hospital, Gothenburg, Sweden

[24]Metabolic Unit, Department of Medical Genetics, CHU & University of Liège, Belgium
[25]Child Neurology and Psychiatry Unit, IRCCS Mondino Foundation, Pavia, Italy
[26]GHUEP, département de neuropédiatrie, Centre de référence neurogénétique mouvement anormaux de l'enfant, Hôpital Armand Trousseau, Paris, France
[27]Pediatric Neurology, The Queen Silvia Children's Hospital, Goteborg, Sweden
[28]Paediatric rheumatology, University College London (UCL) Great Ormond Street Hospital, Institute of Child Health, ARUK Centre for adolescent rheumatology, London, United Kingdom
[29]U.O. Neuropsichiatria infantile, Fondazione IRCCS, Istituto Neurologico Carlo Besta, Milan, Italy
[30]Unit of Child Neurology and Psichiatry, ASST Spedali Civili of Brescia, Italy
[31]Department of Experimental and Clinical Sciences, University of Brescia, Italy
[32]Service de Médecine Infantile, Centre de Référence des maladies métaboliques de Nancy, CHU Brabois Enfants, Unité INSERM NGERE U1256, Nancy, France
[33]Department of Allergy/Immunology, Spectrum Health Helen Devos Children's Hospital, Michigan State University College of Human Medicine, Michigan, USA
[34]Department of Medical Genetics, CHU & University of Liège, Belgium
[35]Service de Neuropédiatrie, Centre Référence des Maladies Lysosomales, Hôpital Trousseau, Paris, France
[36]UF Génétique Médicale et Centre de Référence "Déficiences Intellectuelles", UMR-S975, Groupe Hospitalier Pitié-Salpêtrière, Paris, France
[37]The Liver Unit, Birmingham Women's and Children's Hospital NHS Foundation Trust, Birmingham, United Kingdom
[38]The Rina Mor Institute of Medical Genetics, Metabolic Neurogenetic Service, Wolfson Medical Center, Holon, Israel
[39]Service de pédiatrie, Centre Hospitalier Annecy Genevois, Pringy, France
[40]Department of Paediatric Neurology, Leeds General Infirmary, Leeds, United Kingdom

[41]Pediatric Neurology, Hospital Universitario Donostia, Universidad del País Vasco UPV-EHU, San Sebastian, Spain

[42]Departement de Génétique & Centre de Référence Déficience Intellectuelle de cause rare, GH Pitié-Sapêtrière, Paris, France

[43]INSERM U 1127, CNRS UMR 7225, Sorbonne Universités, UPMC Univ Paris 06 UMR S 1127, Institut du Cerveau et de la Moelle épinière, ICM, F-75013, Paris, France

[44]Service de Neuropédiatrie, GHUEP, Hôpital Armand Trousseau, APHP, Paris, France

[45]Pediatric Neurology Research Group, Hospital Vall d'Hebron – Research Institute (VHIR), Universitat Autonoma de Barcelona, Barcelona, Spain

[46]Institute of Human Genetics, Friedrich-Alexander-Universität Erlangen-Nürnberg (FAU), Erlangen, Germany

[47]Sorbonne Université, GRC n°19, pathologies Congénitales du Cervelet-LeucoDystrophies, CRMR maladies neurogénétiques, Paris, France

[48]Inserm U1141, Groupe Hospitalier HUEP, Service de Neuropédiatrie, Hôpital Trousseau, Paris, France

[49]Developmental Neurology Unit, Fondazione IRCCS Istituto Neurologico Carlo Besta, Milan, Italy

[50]Paediatric Neurology, Starship Children's Hospital, Auckland, New Zealand

[51]Pediatric Neurology Unit, V. Buzzi Children's Hospital, Milan, Italy

[52]Neuropediatría, Hospital Universitari i Pôlitecnic La Fe, Valencia, Spain

[53]West Midlands Regional Clinical Genetics Service and Birmingham Health Partners, Birmingham Women's and Children's Hospitals NHS Foundation Trust, Birmingham, United Kingdom

[54]Department of Paediatric Neurology, Birmingham Women's and Children's Hospitals NHS Foundation Trust, Birmingham, United Kingdom

[55]National Metabolic Service, Starship Children's Hospital, Auckland, New Zealand

[56]Paediatric Neurosciences Research Group, Royal Hospital for Children, Glasgow, United Kingdom

[57]School of Medicine, University of Glasgow, Glasgow, United Kingdom

[58]Department of Brain and Behavioural Sciences, University of Pavia, Pavia, Italy

[59]Paris Descartes University, Sorbonne-Paris-Cité, Institut Imagine, Paris, France

[60]Centre for Genomic and Experimental Medicine, MRC Institute of Genetics and Molecular Medicine, University of Edinburgh, Edinburgh, United Kingdom

*Denotes equal contributions

Corresponding authors

Yanick J. Crow (yanickcrow@mac.com)

Centre for Genomic and Experimental Medicine, MRC Institute of Genetics and Molecular Medicine, University of Edinburgh, Edinburgh, United Kingdom

Sun Hur (Sun.Hur@childrens.harvard.edu)

Department of Biological Chemistry and Molecular Pharmacology, Harvard Medical School, Boston, MA 02115, USA; Program in Cellular and Molecular Medicine, Boston Children's Hospital, MA 02115, USA

Supp. Figure S1. CLUSTAL Omega alignment of IFIH1 homologues

```
H.sapiens           ------------------------------------------------------------
P.troglodytes       ------------------------------------------------------------
M.mulatta           ------------------------------------------------------------
R.norvegicus        ------------------------------------------------------------
M.musculus          ------------------------------------------------------------
C.familiaris        ------------------------------------------------------------
B.taurus            ------------------------------------------------------------
G.gallus            MVHVSFLFELRVLGATFQLFGIGHIGISPCPALPSRGWARLPLRSKQQAKPKAAAGFMLS
X.tropicalis        ------------------------------------------------------------
T.nigroviridis      ------------------------------------------------------------
D.rerio             ------------------------------------------------------------


H.sapiens           ----------------------------------------MSNGYSTDENFRYLISCFRA
P.troglodytes       ----------------------------------------MSNGCSTDENFRYLISCFRA
M.mulatta           ----------------------------------------MSNGYSTDENFRYLISCFRA
R.norvegicus        ----------------------------------------MSTVCSAEDSFRNLISIFRP
M.musculus          ----------------------------------------MSIVCSAEDSFRNLILFFRP
C.familiaris        ---------------------------------------MWSGRPSADQSFRHLLSCFRA
B.taurus            ---------------------------------------MSSDGSSTDKNFCYLISCFRA
G.gallus            ARNGGSLRTADAVPATPTPSAATAAPAPGSSRAGPGAASAAMSEECRDERFLYMISCFRP
X.tropicalis        ----------------------------------------MPQNDSEDARGIYLIECFRA
T.nigroviridis      ----------------------------------------FIMAADSDEIHERLIEYFMP
D.rerio             --------------------------------------MDPNMSSDQDTETRHILDCFRD
                                                                   :     ::  *

H.sapiens           RVKMYIQVEPVLDYLTFLPAEVKEQIQRTVATSGNMQAVELLLSTLEKGVWHLGWTREFV
P.troglodytes       RVKMYIQVEPVLDYLTFLPAEVKEQIQRTVATSGNMQAVELLLSTLEKGVWHLGWTREFV
M.mulatta           RVKMYIQVEPVLDYLTFLPAEVKEQIQRTVATSGNMQAVELLLSTLEKGVWHLGWTREFV
R.norvegicus        RVKMNIQVEPVLDYLVFLPAETKEQILRKVTTCGNTSAAELLLSTLEQGQWPLGWTQMFV
M.musculus          RLKMYIQVEPVLDHLIFLSAETKEQILKKINTCGNTSAAELLLSTLEQGQWPLGWTQMFV
C.familiaris        RVKTYIQVEPVLDYLNFLPEEVKEQIQRKAANAGNLQAAELLLSTLEKGAWPPGWTRQVL
B.taurus            RVKRYIQVEPVLDYLTFLPPEVKEHIQRTAATTGDIQAADLLLNTLERGNWPLGWARMFV
G.gallus            RLKRCIRVQPVLDWLPSLSAEEKDKVRAAALQRGEVEGAEELLCAVERGRRDPGWFTEFL
X.tropicalis        RLVRYIQAVPVLDHLTWLGRDIREQVVSKAQNQGEQDAARLLLDRIVRGPREPGWFEAFV
T.nigroviridis      RLRNLIVVQQVLDFLDFIEPDQKERIRQRNISRGNLAAADALITAVTQKPHQAGWFRAFV
D.rerio             RLKRLIQVEPLLDLLDFLEPDKKERIKAKVASEGNINAVSLLINEIMRAELQQGWSRTLV
                    *:   * .  :** *  :  : ::::       *:  ..  *:  : :     **   .:

H.sapiens           EALRRTGSPLAARYMNPELTDLPSPSFENAHDEYLQLLNLLQPTLVDKLLVRDVLDKCME
P.troglodytes       EALRRTGSPLAARYMNPELTDLPSPSFENAHDEYLQLLNLLQPTLVDKLLVRDVLDKCME
M.mulatta           EALRRAGSPLAARYMNPELTDLPSPSFENAHDECLQLLNLLQPTLVDKLLVRDVLDKCME
R.norvegicus        EALEHSGNPLAARYVKPSLTDLPSPSSETAHDECLHLLNLLQPTLVGKLLINDVLDTCSE
M.musculus          EALEHSGNPLAARYVKPTLTDLPSPSSETAHDECLHLLTLLQPTLVDKLLINDVLDTCFE
C.familiaris        VALQSAGSVLASRYLNPELADLPSPSAENAHDQCLQLLHLLQPTLVDRLLVKDVLDKCVE
B.taurus            EALRQAGNPLAARYVNPELTDLPSPSSENAHDECLQLLNLLQPTLVDKLLVADVLDKCVE
G.gallus            LALKKGGCDLAACYVNPS--QLPSPQEEDDHDLCVHLVQLLHGTLVDNMQTRQVAEKCLE
X.tropicalis        RALKDSHCTQAAAYLSE---GRPTPSLEATWDYYEQLLIVLYPELIAKIDPKETAPLCRR
T.nigroviridis      DALEQSGCGHAADYIQN---KIPEPEVEAENDYCIRLIQLLSPSLVD-MKTSAVCVQCLS
D.rerio             TALETVGCGHAARYVRN---RPPEPDEEAEHDSCVRLIDLLHLSLVN-MKTRDVCVQCVT
                     **.      *: *:       * *. *   *   :*: :*   *:  :    .   *

H.sapiens           EELLTIEDRNRIA-AAENNGNESGVRELLKRIVQK-ENWFSAFLNVLRQTGNNELVQELT
P.troglodytes       EELLTIEDRNRIA-AAENNGNESGVRELLKRIVQK-ENWFSAFLNVLRQTGNNELVQELT
M.mulatta           EELLTIEDRNRIA-AAENNGNESGVRELLKRIVQK-ENWFSAFLDVLRQTGNDELVQELT
R.norvegicus        KGLVTVEDRNRIS-AAGNSGNESGVRELLRRIVQK-ENWFSTFLDVLRQTGNDALVQELT
M.musculus          KGLLTVEDRNRIS-AAGNSGNESGVRELLRRIVQK-ENWFSTFLDVLRQTGNDALFQELT
C.familiaris        KKLLTDEDRDRIS-AAENNGNQSGVRELLKRIVQK-ENWFSLFLTVLNQTENYALVQELT
B.taurus            EKLLTIEDRNRVS-AAENNGNEAGVRELLKRIVQK-ENWFSTFLTILRQTGNDALAREFT
G.gallus            LGIFQEEDLVGIETVIESRGNRDGARELLSRIVQK-KDWFSQFLVALRETQHESLADDLS
X.tropicalis        EEICSDEDVNVISNVTDQHGNQQGSRELLNRIIKK-QDWFSKFLTVLRTMGQNSLADYLT
T.nigroviridis      QDLITEDDSENIEAATRNQGAKAGARELLGRIVRGRPGWFSKFLDILLKTEHKNLYSELT
D.rerio             LGLLTQEDQENIMTATGNNGNINGARLLLKRLVKNEAGWFSKFLQALEDTEHRDLMRELR
                      :   :*   :  .  . *   * * ** *:::    *** **  *    .  *   :
```

```
H.sapiens            GSDCSESNAEIENLS--QVDGPQVEEQLLSTTV--QPNLEKEVWGMENNSSESSFADSS-
P.troglodytes        GSDCSESNAE--------------------------------------------------
M.mulatta            GTDCSESNAEIENLS--QDDGPQVEEQLLSTTV--QPNLEKEVWGMENNSSESSFADSS-
R.norvegicus         GVSCPEESTDLDNAS--HKDRPAANEPLLPAID--ALSLETEAWTIEDTSPEASFADSS-
M.musculus           GGGCPEDNTDLANSS--HRDGPAANECLLPAVD--ESSLETEAWNVDDILPEASCTDSS-
C.familiaris         GTTCFESKEETENLS--QEDGPEVKAAALLAMS--QPSPEKEGWDMENNSLESSLLDSS-
B.taurus             GTDCCEGSTESENLS--QEDGLEVKEPLLLATD--QPNLE--VLDIENSSLESSFADSS-
G.gallus             GNTGGTEDKDYELKNNTGKKTEAASQPVYVTEDLKQQNLD-DSFVRESSVLETSVGKNS-
X.tropicalis         GSNNDENAESSGARPT--VEGADD-SSSVADIKMQHHELGSHDVKCENNLAESSFAGSN-
T.nigroviridis       GGSPDCNKQDRQIKY--LYEGPPAEQPSSVEVV--QPDSD-ERLSLGQLHGSPDLSPAET
D.rerio              GEPCEGDEGMS-VD----SSDAQ------AEVK--DTGAV-RRNSM--SSPPPSFTPNSS
                     *

H.sapiens            -VVSES---DTSLAEGSVSCLDESLGH---NS-----NMGSDSGTMGSDSDEENV--AAR
P.troglodytes        -----S---DTSLAEGSVSCLDESLGH---NS-----NMGSDSGTMGSDSDEENV--AAR
M.mulatta            -VVSES---DTSLAEGSVSCLDESLGH---NS-----NMGSDSGTMGSDSDEENV--AAR
R.norvegicus         -VTTES---DTSLAEGSVSCFDESLGH---NS-----NMGRDSGTMGSDSDEDTIMGTKR
M.musculus           -VTTES---DTSLAEGSVSCFDESLGH---NS-----NMGRDSGTMGSDSDES-VIQTKR
C.familiaris         -VVSES---DTSLAEGSVSCLDESLGH---NS-----NMGSDSGTMGSDSDEENV--AER
B.taurus             -IVSES---DTSLAEGSVSCLDESLGH---NS-----NMGSDSGTMGSDSDDENV--AQR
G.gallus             -VISES----VAVGDASVSNSNE--------------NLGQSSTTSDSALGED--EAEGR
X.tropicalis         -ATSDL---DTSSPELYCSADIESLEI---SDSALLRWMQT--NTITYLADEQEETTASR
T.nigroviridis       EQMSQSLTETLSLTPGNLCKPSVLVGSK-SQSGRHLRILGCSSS--------QPNGTRAG
D.rerio              LQ-SEDLDDSV---------DSSLLGVDAGNQGVEMYEDGEEKEEK----DLKEDEDDDS
                                                                         .
                                                  T331R
                                                  T331I R337G
H.sapiens            ASPEPELQLRPYQMEVAQPALEGKNIIICLPTGSGKTRVAVYIAKDHLDKKKKASEPGKV
P.troglodytes        ASPEPELQLRPYQMEVAQPALEGKNIIICLPTGSGKTRVAVYIAKDHLDKKKKASEPGKV
M.mulatta            ASPEPELQLRPYQMEVAQPALEGKNIIICLPTGSGKTRVAVYIAKDHLDKKKKASEPGKV
R.norvegicus         ASPKPELQLRPYQMEVAQPALDGKNIIICLPTGSGKTRVAVYITKDHLDKKKQACESGKV
M.musculus           VSPEPELQLRPYQMEVAQPALDGKNIIICLPTGSGKTRVAVYITKDHLDKKKQASESGKV
C.familiaris         ASPEPELHLRPYQMEVAQPALEGKNIIICLPTGSGKTRVAVYIAKDHLDKKKKASEPGKV
B.taurus             ASPEPELNLRPYQLEVAQPALEGKNIIICLPTGSGKTRVAVYIAKDHLDKK-KASEHGKV
G.gallus             ASPEPDLTLRDYQMEVAKPALNGENIIICLPTGSGKTRVAVYITKDHLDKKRKASEQGKV
X.tropicalis         ASPVPQITLRNYQMEVAKPALEGKNIIICLPTGSGKTRVAVYITREHLCKRREEGRLAKA
T.nigroviridis       DEESEDIDLWDYQMEVARPALEGENIIICLPTGRGKTRVAVYVAKKHLESRKAKGKIGKV
D.rerio              SASKREIKLRDYQMEVARPALEEKNIIVCLPTGSGKTRVAVFITKEHLERKQRMGQKGKV
                          :: *  **:***:***: :***:***** *******::::.**  :    . .*.
                                                      D393A
                                               G389R  D393V
H.sapiens            IVLVNKVLLVEQLFRKEFQPFLKKWYRVIGLSGDTQLKISFPEVVKSCDIIISTAQILEN
P.troglodytes        IVLVNKVLLVEQLFRKEFQPFLKKWYRVIGLSGDTQLKISFPEVVKSCDIIISTAQILEN
M.mulatta            IVLVNKVLLVEQLFRKEFKPFLKKWYRVIGLSGDTQLKISFPEVVKSCDIIISTAQILEN
R.norvegicus         IVLVNKVMLAEQLFRKEFNPFLKKWYRIIGLSGDTQLKISFPEVVKSYDVIISTAQILEN
M.musculus           IVLVNKVMLAEQLFRKEFNPYLKKWYRIIGLSGDTQLKISFPEVVKSYDVIISTAQILEN
C.familiaris         IVLVNKVPLVEQLFREEFEPFLKKWYHTIGLSGDTQLKISFPEIVKTYDVIISTAQILEN
B.taurus             MVLVNKVPLVEQLFRKEFKPFLKKWYHVTRLSGDTQLKITFPEVVKSHDVIISTAQILEN
G.gallus             IVLVNKVPLVEQHLRKEFNPFLKRWYQVIGLSGDSELKISFPEVVKRYDVIICTAQILEN
X.tropicalis         IVLVNKVPLVEQHYRREFYPFLKDHYQVTKISGDSQLKNSFHKVVQEHDVVICTAQILEN
T.nigroviridis       VVLVNQIPLVEQHYATEFLPFLKHTYKVERVSGDSQLKISFTDTVRKNDVIICTAQILEN
D.rerio              VVLVNKVPLVEQHYKAEFGRFLKHQYSVERVSGASQLKISFPQIIEKNDIIICTAQILEN
                     :****:: *.**    **  :**  *    :** ::** :* . :.  *::*.*******

                                           E444G N449K
H.sapiens            SLLNLENGEDAGVQLSDFSLIIIDECHHTNKEAVYNNIMRHYLMQKLKNNRLKKENKPVI
P.troglodytes        SLLNLENGEDAGVQLSDFSLIIIDECHHTNKEAVYNNIMRRYLMQKLKNNRLKKENKPVI
M.mulatta            SLLNLENGEDAGVQLSDFSLIIIDECHHTNKEAVYNNIMRRYLMQKLKNNRLKKENKPVI
R.norvegicus         SLLNLESGEDDGVQLSDFSLIIIDECHHTNKEAVYNNIMRRYLKQKLKNHKLKKQNKPTI
M.musculus           SLLNLESGDDDGVQLSDFSLIIIDECHHTNKEAVYNNIMRRYLKQKLRNNDLKKQNKPAI
C.familiaris         SLLNSEKGEDDGVQFSDFTLIIIDECHHTNKEAVYNNIMRRYLKQKLKNNKLKKEYKPVI
B.taurus             SLLNSEEGEDDGIELSDFSLIIIDECHHTNKEAVYNNIMRRFLKQKLKNNKLKKENKPVI
G.gallus             SLLNAT-EEDESVRLSDFSLIIIDECHHTQKEGVYNNIMRRYLKEKIKNRKQAKENKPLI
X.tropicalis         SLIQAAEDEEEGVQLSDFSLIIIDECHHTQKDAVYNNIMIRYIKKKMQNKRNSKMEKAQV
T.nigroviridis       YLERSRTGEDEGVNLSDLSLIVIDECHHTQKGGVYNQIMVRYLMQKHKNIKLRKEQKPTA
D.rerio              SLAKAKNGDEDGIELSQFTLMVIDECHHTKKGGVYNHIMIRYLKQKNRNQLLKKQDKTLV
                      * .    :: .:.:*:::*::*******:* .***.** ::: :* :*    *  *
```

```
                          A489T  G495R
H.sapiens           PLPQILGLTASPGVGGATKQAKAEEHILKLCANLDAFTIKTVKENLDQLKNQIQEPCKKF
P.troglodytes       PLPQILGLTASPGVGGATKQAKAEEHILKLCANLDAFTIKTVKENLDQLKNQIQEPCKKF
M.mulatta           PLPQMLGLTASPGVGGATKQAKAEEHILKLCANLDAFTIKTVKENLDQLKNQIQEPCKKF
R.norvegicus        HLPQILGLTASPGVGAAKKQSEAEKHILNICANLDAFTIKTVKENLSQLKHQIKEPCKKF
M.musculus          PLPQILGLTASPGVGAAKKQSEAEKHILNICANLDAFTIKTVKENLGQLKHQIKEPCKKF
C.familiaris        PLPQILGLTASPGVRGAKRQAEAQQHILKICANLDACTIITVKENINQLKGQMKEPCKKF
B.taurus            PLPQIVGLTASPGVGGAKKQAKAEEHILKICANLDAVTIKTVKQNINLLKEQIKEPCKKF
G.gallus            PQPQILGLTASPGVGGARSNSKAEEHILKICANLDACRIMTVKEHASQLKNQVKEPFKKT
X.tropicalis        PLPQILGLTASPGVGGAKNIKKSEEHILRICANMDAFKIMTVQENAEQLRKQVKDPYKEV
T.nigroviridis      PLPQILGLTASPGVGSATKMAKAVEHILRICANLDASRIMTLPPGE----L-KRDSKKDV
D.rerio             PIPQILGLTASPGVGGAVSQQMAEEHILQICANLDAFTIKTKTFEE----EEAKTPFKRI
                      **::******** .*     : :***.:***:**  * *            :   *

                                                         I583V
H.sapiens           AIADATREDPFKEKLLEIMTRIQTYCQMSPMSDFGTQPYEQWAIQMEKKAAKEGNRKERV
P.troglodytes       AIADATREDPFKEKLLEIMTRIQTYCQMSPMSDFGTQPYEQWAIQMEKKAAKEGNRKERV
M.mulatta           AIADDTREDPFKEKLLEIMTRIQTYCQMSPMSDFGTQPYEQWAIQMEKKAAKEGNRKDRV
R.norvegicus        VIADDTRENPFKEKLLEIMASIQTYCQKSPLSDFGTQHYEQWAIQMEKKAAKDGNRRDRV
M.musculus          VIADDTRENPFKEKLLEIMASIQTYCQKSPMSDFGTQHYEQWAIQMEKKAAKDGNRKDRV
C.familiaris        AIADDTREDPFKDKLLEIMNKIQSFCQMSPMSDFGTQPYEQWTIQMEKKAAREGNRKDRV
B.taurus            VIADDTKKDPFKDKLLEIMTKIQTFCQINPMSDFGTQPYEQWLIQMEKKAAKEGNRKDRV
G.gallus            VIADDKRRDPFRERIIEIMQDIQKYCQLYPKSEFGSQPYEQWVIREERRAAKEEKRKERV
X.tropicalis        KISDEKKKNPFGDKLKEIMGKIEEYSKLYPTSDHGSQSYEQWVIQTDKTAAKEGKRKEHV
T.nigroviridis      IPVEDRKQDPFGNVIKKLMNTIHNHAHLRPNSDLGSQTYEQWVVQMEREAALAGDHKVRA
D.rerio             AKAEERKEDPFGDVIKKIMDEIHTHADLQPLCEPGTQNYEQWVVQKEQNAAKEENQNVRV
                       :  :.:** : : ::*  *. ...  * .: *:* **** :: :: **   .:. :.

H.sapiens           CAEHLRKYNEALQINDTIRMIDAYTHLETFYNEEKDKKFAVIEDDSDEGGDDEYC--DGD
P.troglodytes       CAEHLRKYNEALQINDTIRMIDAYTHLETFYNEEKDKKFAVIEDDSDEGGDDEYC--DGD
M.mulatta           CAEHLRKYNEALQINDTIRMIDAYNHLETFYNEEKDKKFAVIEDDSDEGGDDEYC--DGD
R.norvegicus        CAEHLRKYNEALQINDTIRMIDAYSHLETFYTDEKEKKFAALNDSDES--DDDEASSCHD
M.musculus          CAEHLRKYNEALQINDTIRMIDAYSHLETFYTDEKEKKFAVLNDSDKS--D-DEASSCND
C.familiaris        CAEHLRKYNEALQINDTIRTIDAYNHLEAFYNDETEKKLAVQEGDSDESGEDGDGDGDGD
B.taurus            CAEHLRKYNEALQINDTIRMIDAYNHLETFYNDEKEKKFAVLLGDDSDESD---D--NGD
G.gallus            CAEHLKKYNDALQINDTIRMVDAYNHLNNFYKELKRRKTAESDDD---------------
X.tropicalis        CAEHLRKYNDALQINDTIRMTDSLIHLRKFYEEEKKRKILLNEGS---------------
T.nigroviridis      CAEHLRQYNEGLGLSNTFRMWDALNFLSKYHEEEMKKKTAPD------------------
D.rerio             CAEHLRQYNEALHQSNTIRMSDAFRFLDKYHNEELKTKSSPD------------------
                    *****::**:.*  .:*:*  *:  .*  :: :    *

H.sapiens           EDEDDLKKPLKLDETDRFLMTLFFENNKMLKRLAENPEYENEKLTKLRNTIMEQYTRTEE
P.troglodytes       EDEDDLKKPLKLDETDRFLMTLFFENNEMLKRLAENPEYENEKLTKLRNTIMEQYTRTEE
M.mulatta           EDEDDLKKPLKLDETDRFLMTLFFENNKMLKKLAENPEYENEKLTKLRNTIMEQYTRTEE
R.norvegicus        QLKGNVKKSLKLDETDEFLMNLFFDNKKMLKKLAENPKYENEKLIKLRNTILEQFTRSEE
M.musculus          QLKGDVKKSLKLDETDEFLMNLFFDNKKMLKKLAENPKYENEKLIKLRNTILEQFTRSEE
C.familiaris        EAEDDEKRPLRLDRTDRFLMELFWENKRMLKKLAQNPKHENEKLIKLRNTIMEQFTRTEE
B.taurus            DDVGDGKPPLKLHETDDFLISLFWGNKKKLKKLAQNPEHENEKLIKLRNTIMEQYSRTEG
G.gallus            ----EEPLVSKQDETDEFLMRLFHAKKKQLKELARKPEYDNEKLMKLRNTLMEEFTKTEE
X.tropicalis        ----EHVAPLNIEETDRFLIDLFYDNEKELTAIAKNPKYENENLYALRSSLLEEFTRNGQ
T.nigroviridis      -----EEHVIQITDTERFLFNLFKEYKEELQTLANNPEYENKSLSKLKTKVLQEFSTRLD
D.rerio             -----EEGNITITDTERFLFTLFKDKKAKLQELMGKPQYENNNLAQLKTIILKEFSTREK
                                  *: **: **   :  *  :  :*:::*:.*  *:. ::::::

                 A719V R720Q                                           E773Q
H.sapiens           SARGIIFTKTRQSAYALSQWITENEKFAEVGVKAHHLIGAGHSSEFKPMTQNEQKEVISK
P.troglodytes       SARGIIFTKTRQSAYALSQWITENEKFAEVGVKAHHLIGAGHSSEFKPMTQNEQKEVISK
M.mulatta           SARGIIFTKTRQSAYALSQWITENEKFAEVGVKAHHLIGAGHSSEFKPMTQNEQKEVISK
R.norvegicus        SSRGIIFTKTRQSTYALSQWIMENEKFAEVGVKAHHLIGAGHSSEVKPMTQTEQKEVISK
M.musculus          SSRGIIFTKTRQSTYALSQWIMENAKFAEVGVKAHHLIGAGHSSEVKPMTQTEQKEVISK
C.familiaris        PARGIIFTKTRQSAFALSQWITENKKFAEVGVKAHHLIGAGHSSEFKPMTQNEQKEVISK
B.taurus            SARGIIFTKTRQSAYALSQWIIENEKFSEVGVKAHHLIGAGHSSEFKPMTQNEQKEVISK
G.gallus            -PRGIIFTKTRQSALALYHWIMDNPKFEEVGIKAHFLIGAGHNSETKPMTQNEQREVIDK
X.tropicalis        -ARGIIFTKTRQSAVALNQWISDNEKFTEVGIRSSYLIGAGHNSDFKPMTQNEQKQIIHK
T.nigroviridis      -ARGIIFTKTRRGAIALTQWIRENSKFADMDVKPAYVIGGGDQSVVKPMTAAEQKDVLNK
D.rerio             -ARGIIFTQTRLSAIALCQWIEENPKFDEVGVRASYLIGGGDQSVVKPMTAAEQKDVLKR
                      ******:** .: ** :** :* ** :: ::  .:**.*..*  ****  **:::: :
```

```
                    R779L                     N802D
                    R779C G781E                I803F                       T829S
                    R779H                                     R822Q R824K
H.sapiens              FRTGKINLLIATTVAEEGLDIKECNIVIRYGLVTNEIAMVQARGRARADESTYVLVAHSG
P.troglodytes          FRTGKINLLIATTVAEEGLDIKECNIVIRYGLVTNEIAMVQARGRARADESTYVLVAHSG
M.mulatta              FRTGKINLLIATTVAEEGLDIKECNIVIRYGLVTNEIAMVQARGRARADESTYVLVAHSG
R.norvegicus           FRTGEINLLIATTVAEEGLDIKECNIVIRYGLVTNEIAMVQARGRARADESTYVLVTSSG
M.musculus             FRTGEINLLIATTVAEEGLDIKECNIVIRYGLVTNEIAMVQARGRARADESTYVLVTSSG
C.familiaris           FRTGKINLLIATTVAEEGLDIKECNIVIRYGLVTNEIAMVQARGRARADESTYVLVAHSN
B.taurus               FRTGKINLLIATTVAEEGLDIKECNIVIRYGLVTNEIAMVQARGRARADESTYVLVAQSG
G.gallus               FRGGSINLLIATTVAEEGLDIKECNIVIRYGLVTNEIAMVQARGRARADESTYALVASSG
X.tropicalis           FSTGELNLLVATSVAEEGLDIKECNVVIRYGLVTNEIAMVQARGRARADDSSYVLVAPSS
T.nigroviridis         FRNGEVNLLIATSVAEEGLDIPKCNFVIRYGHVANEISMIQTEGRGRAEDSSYTVVDVKN
D.rerio                FRTGEINLLIATTVAEEGLDIAECNVVIRYCLVTNEVAMIQARGRGRAEDSSYTLVAEAG
                       *  *.:***:**:********  :**.****   *:**::*:*:.**.**::*:*.:*

                              D848E  M854K
H.sapiens              SGVIEHETVNDFREKMMYKAIHCVQNMKPEEYAHKILELQMQSIMEKKMKTKRNIAK-HY
P.troglodytes          SGVIERETVNDFREKMMYKAIHCVQNMKPEEYAHKILELQMQSIMEKKMKTKRNIAK-HY
M.mulatta              SGVIERETVNDFREKMMYKAIHCVQNMKPEEYAHKILELQMQSIMEKKMKTKRSIAK-HY
R.norvegicus           SGVTEREIVNDFREKMMYKAINRVQNMKPEEYAHKILELQVQSILEKKMKVKRSIAR-QY
M.musculus             SGVTEREIVNDFREKMMYKAINRVQNMKPEEYAHKILELQVQSILEKKMKVKRSIAK-QY
C.familiaris           SGVIEREIVNDFREKMMYKAIDHVQNMNPEEYAHKILELQMQSIMEKKMKIKRSAAK-CY
B.taurus               SGVVERETVNDFREKMMYKAIDRVQNMKPEEYAHKILELQMQSIMEKKMKTKRSIAK-QF
G.gallus               SGAVEREDVNIFRENMMYKAIRRVQEMPPEEYLNKIQDFQLQSIVEKQMKAKRDQRK-TY
X.tropicalis           SGAIERDSVNVYKEEMMHKAIAKVQKMDRATYIDKIEEFQAQTIMEKKVKAKKAIHK-VY
T.nigroviridis         SGVAEKETVNEYRKNMMDKAIEKIGALKHADYDKQIQEFQMQAIMEYMLRMKAKKQEDIK
D.rerio                SGVAERESVNEYREKMMSKAIAKVCKMNRADYEKKIMEFQIQAIMEEKVKTKKKQQKGMM
                       **. *:: ** ::::** ***  :  :    *  .:*  ::*  *:*:*   ::  *    .

                                                                              I956V
H.sapiens              KNNPSLITFLCKNCSVLACSGEDIHVIEKMHHVNMTPEFKELYIVRENKALQKKCADYQI
P.troglodytes          KNNPSLITFLCKNCSVLACSGEDIHVIEKMHHVNMTPEFKELYIVRENKALQKKCADYQI
M.mulatta              KNNPSLITFLCKNCSVLACSGEDIHVIEKMHHVNMTPEFKELYIVRENKALQKKCADYQT
R.norvegicus           NSDPSLITLLCKNCSTLVCSGENIHVIEKMHHVNMTPEFKGLYIVRENKALQMKFADYQT
M.musculus             NDNPSLITLLCKNCSMLVCSGENIHVIEKMHHVNMTPEFKGLYIVRENKALQKKFADYQT
C.familiaris           KENPSLINFLCKNCSVLACSGEDIHVIEKMHHVNMTPVFKELYIVRENKALRKKFIDYQT
B.taurus               KGKPSLINFLCKNCGVPACSGEDIHVIEKMHHVNMTPEFKKLYLVRGNKALQTMCVDYQT
G.gallus               KKNPSLITFLCKNCHKLICSGEDIQVIENMHHVSVKKDFQHLYHKRENRTLQDKHADYQT
X.tropicalis           QSNPSLVTFHCKKCSKQACCGTDIQVIATAHHVNTTPKFKTLYSKGPNKTLQEKFADYQI
T.nigroviridis         NENPSNVKFSCRSCSQEVCTGRDIEIMANIHRVNVTPQFRELFILKENTKLKNSLLDYET
D.rerio                KEPPSKISLSCRQCSVFVCSGEDIEIIEKMHHVNVTKQFRKLFIVRENASLQERLLDYET
                       :  ** :.: *:.*    * * :*.:: . *:*. .   *: *:    *   *:    **:

                                        L979W
H.sapiens              NGEIIC-KCGQAWGTMMVHKGLDLPCLKIRNFVVVFKNNS-TKKQYKKWVELPITFPNLD
P.troglodytes          NGEIIC-KCGQAWGTMMVHKGLDLPCLKIRNFVVVFKNNS-TKKQYKKWVELPITFPNLD
M.mulatta              NGEIIC-KCGQAWGTMMVHKGLDLPCLKIRNFVVVFKNNS-TKKQYKKWVELPITFPNLD
R.norvegicus           NGEIIC-KCGQAWGTMMVHKGLDLPCLKIRNFVVNFKNNS-SKKQYKKWVELPIRFPDLD
M.musculus             NGEIIC-KCGQAWGTMMVHKGLDLPCLKIRNFVVNFKNNS-PKKQYKKWVELPIRFPDLD
C.familiaris           NGEIICKMCGQAWGTMMVHKGLDLPCLKIKNFVVIFKNNT-SKKQYKKWVELPIAFPDLD
B.taurus               NGEIICNKCGQAWGTMMVHKGLDLPCLKIKNFVVVFQNNL-PKKQYKKWVELPITFPDLN
G.gallus               NVEIICKDCGQVWGNMMVYRGLDLPCLKIRNFVVAFEDKKTTKEIFKKWGELPIIFPDFD
X.tropicalis           NGDIICKECGKTWGTTMVHKGIEVPCLQIRNFVVKYDDKKMTKDTYDKWSELPIKFPSFT
T.nigroviridis         NGYIACGKCGQKWGSMMHFRGIQCPCLHVKHFVVTINGKK--ISKCQQWMDLPVRLSEFD
D.rerio                NGVIACKKCGQQWGSMMLYRSTECPCLHIKNFVVTYGSK---KKTFSKWRELSISFPAFD
                       *  * *  **: **. *  .:. : ***:::.***    :    .  .:* :* : :  :

H.sapiens              YSECCLFSDED---------
P.troglodytes          YSECCLFSDED---------
M.mulatta              YSECCLFSDED---------
R.norvegicus           CSEYCLYSDED---------
M.musculus             YSEYCLYSDED---------
C.familiaris           YSEYCLFSDED---------
B.taurus               YSEYCLFSDED---------
G.gallus               YASHCPSSDED---------
X.tropicalis           YSLP--DSDED---------
T.nigroviridis         YAEHCLFKYSY---------
D.rerio                YTQHADLLVEDSEDEDMDTN
                        :       .
```

IFIH1 homologs were identified on Ensembl and aligned using CLUSTAL Omega. Amino acids altered by IFIH1 mutations are highlighted by a red box. H.Sapiens, Homo sapiens (ENSP00000263642); P.troglodytes, Pan troglodytes (ENSPTRG00000012582); M.mulatta, Macaca mulatta (ENSMMUG00000003202); R.Norvegicus, Rattus norvegicus (ENSRNOG00000006227); M.Musculus, Mus musculus (ENSMUSG00000026896); C.Familiaris, Canis familiaris (ENSCAFG00000010438); B.Taurus, Bos Taurus (ENSBTAG00000008142); G.gallus, Gallus gallus (ENSGALG00000011089); X.Tropicalis, Xenopus tropicalis (ENSXETG00000013176); T.nigroviridis, Tetraodon nigroviridis (ENSTNIG00000016500); D.Rerio, Danio rerio (ENSDARG00000018553). Homology to the human IFIH1 reference sequence (ENSP00000263642): human–P. troglodytes, 100%; human–M. mulatta, 98%; human–B. torus, 84%; human–C. familiaris, 83%; human–M. musculus, 80%; human–R. norvegicus, 80%; human–G. gallus, 56%; human–X. tropicalis, 55%; human–D. rerio, 49%; human–T. nigroviridis, 44%.

Supp. Figure S2. **Interferon beta (IFN-ß) reporter activity of Flag-tagged wild-type (WT) and mutant *IFIH1* with and without stimulation with polyI:C in HEK 293T cells.**

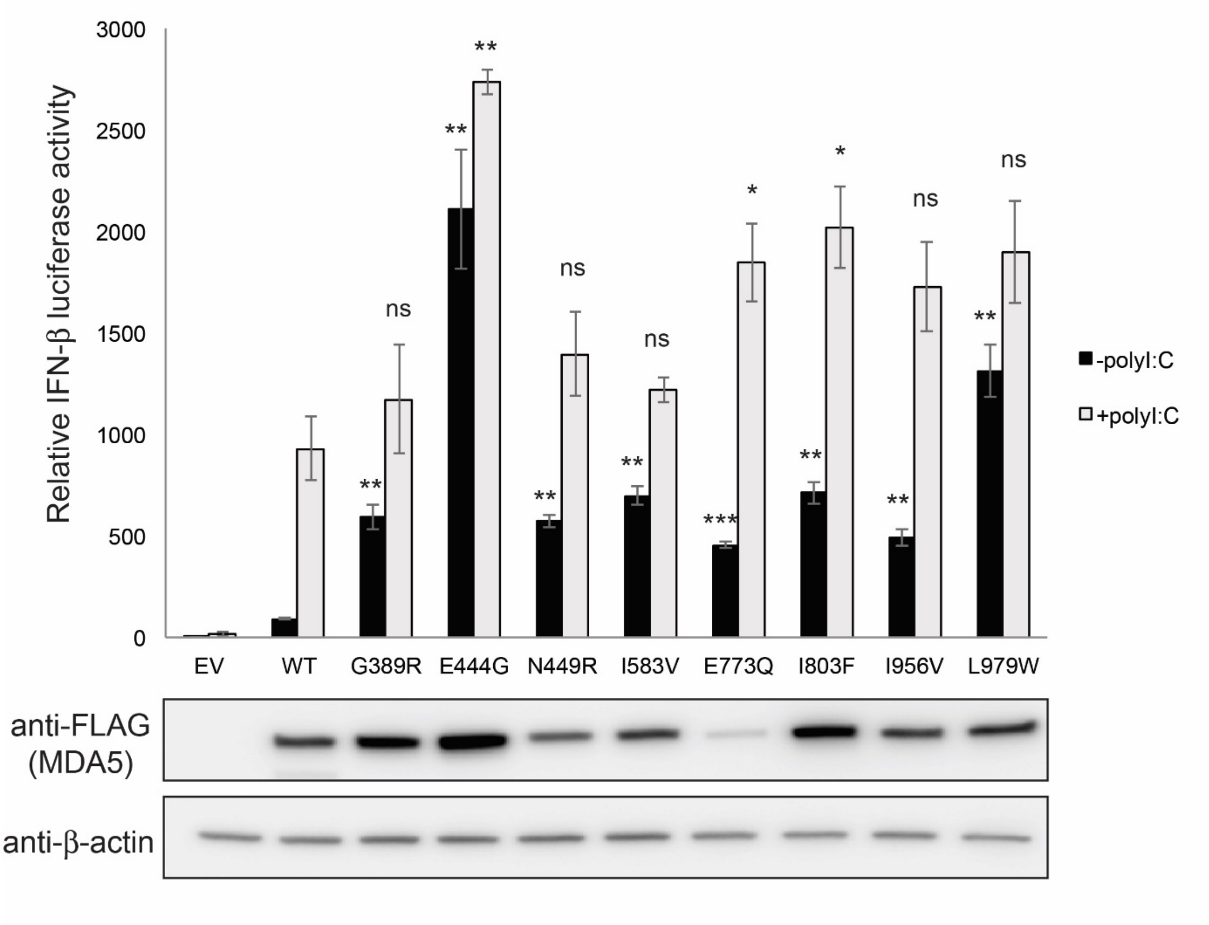

Error bars represent the standard deviation of three independent experiments. The IFIH1 variants p.Gly389Arg, p.Asn449Lys, p.Ile583Val, p.Ile803Phe and p.Ile956Val are predicted as benign using *in silico* programs. The other three variants (p.Glu444Gly; p.Glu773Gln; p.Leu979Trp) were tested in view of the novelty of the mutation and / or an absence of interferon stimulated gene testing in a variant carrier. EV indicates empty vector. Statistical significance was determined by two-tailed, unpaired Student's t-test with *, ** and *** indicating P values <0.05, <0.01, and <0.001 respectively. ns indicates not significant. All variants showed significantly higher basal signaling activity compared to WT in the absence of polyI:C. Expression levels of individual constructs were tested by western blotting. While the level of E773Q (p.Glu773Gln) is significantly lower than WT IFIH1 or other variants, this

does not affect our interpretation that E3773Q (p.Glu773Gln) is a gain-of-function mutation.

Annotated p values are in comparison to the wild type (WT) value.

Supp. Figure S3. Interferon scores in *IFIH1*-mutation positive individuals as measured in the Crow laboratory.

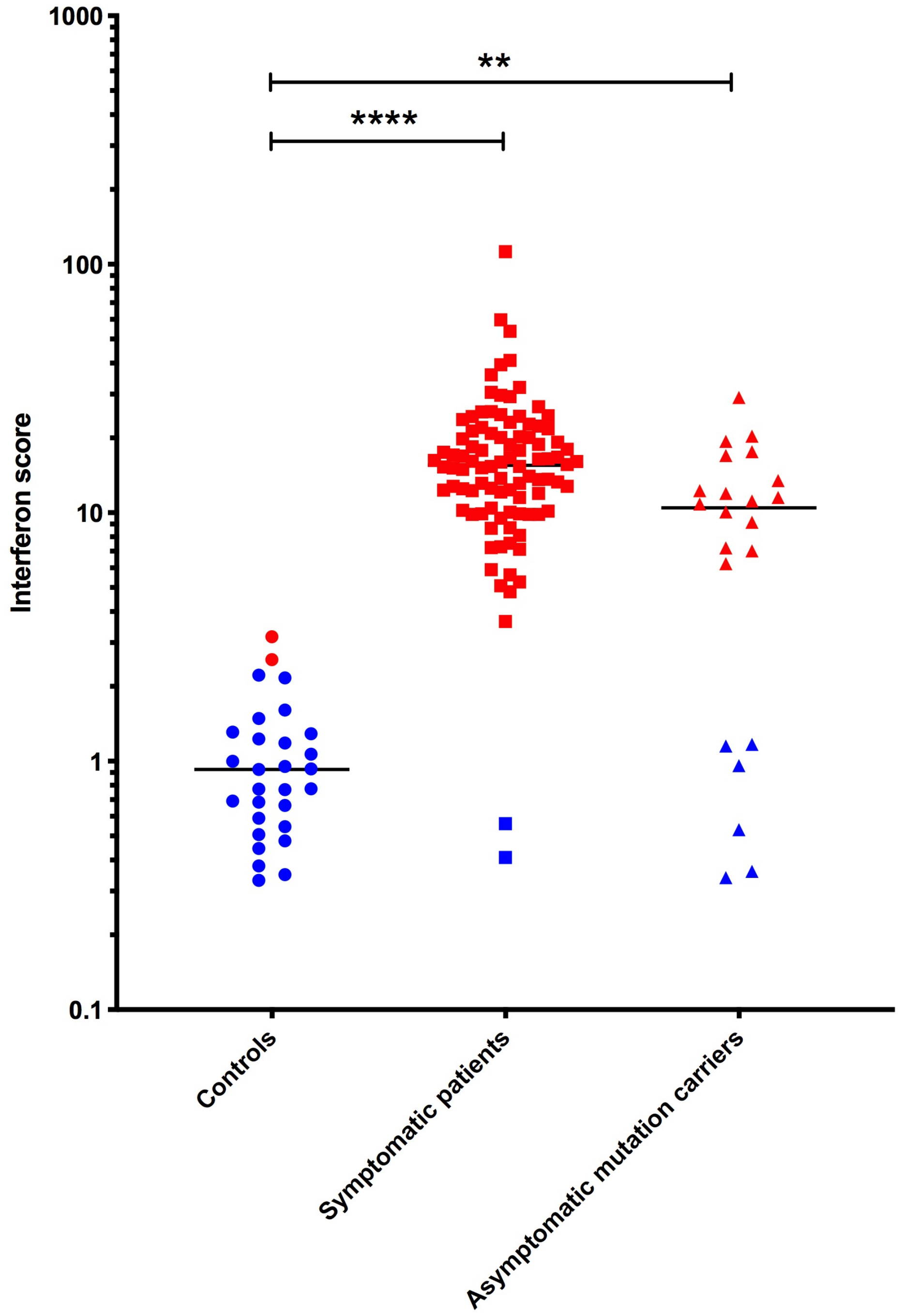

Red denotes elevated score (> 2.44), blue denotes a result within the normal range of 29 controls as previously published (Rice et al. Lancet Neurol 12;1159-69).

Supp. Table S1. Details of six putative mutations predicted as benign by *in silico* analysis

cDNA change	Protein change	Mutated families (de novo inheritance; or, number of symptomatic and non-penetrant individuals where familial)	Associated phenotypes ('/' within family)(';' between families)	Upregulated interferon stimulated gene expression	*In vitro* enhancement of interferon signalling	gnomAD frequency	SIFT	Polyphen2	CADD score	Varcards
c.1165G>A	p.Gly389Arg	AGS848 (2; 1)	AGS/SP/CNP	Yes (four variant-positive individuals, all demonstrating a positive interferon signature on at least two occasions: Crow laboratory)	Yes (this paper)	Novel	Tolerated 0.88	Benign 0.108	5.325	01:23
c.1347C>G	p.Asn449Lys	AGS1001 (de novo)	SP	Yes (interferon score positive on both occasions tested: Crow laboratory)	Yes (this paper)	Novel	Tolerated 0.64	Benign 0.163	13.91	03:23
c.1747A>G	p.Ile583Val	AGS2369 (de novo)	AGS	Yes (interferon score positive on both occasions tested: Crow laboratory)	Yes (this paper)	Novel	Tolerated 0.48	Benign 0.00	0.573	5.23
c.2407A>T	p.Ile803Phe	LD_1488.0 (de novo)	AGS	Yes (interferon score positive on three occasions tested: Vanderver laboratory)	Yes (this paper)	Novel	Tolerated 0.24	Benign 0.043	11.8	04:23

c.2544T>G	p.Asp848Glu	AGS531 (3; 2)	SP-ICC/CNP	Yes (four variant-positive individuals, all demonstrating a positive interferon signature on at least two occasions: Crow laboratory)	Yes (Ruaud et al. 2018)	Novel	Tolerated 0.4	Benign 0.004	10.08	02:23
c.2866A>G	p.Ile956Val	AGS1430 (2; 1)	SP-ICC/CNP	Yes (two variant-positive individuals demonstrating a positive interferon on every occasion (four and three times) tested; one variant-positive individual, an asymptomatic grandfather to the proband, with a normal interferon score on two occasions tested: at the age of 69 years)	Yes (this paper)	Novel	Tolerated 0.77	Benign 0.004	3.576	06:23

IFIH1 mutation annotation based on the reference cDNA sequence NM_022168.2

AGS: Aicardi-Goutières syndrome; CLL: Chilblain-like lesions; CNP: Clinical non-penetrance; ICC: Intracranial calcification; SP: Spastic paraparesis

Supp. Table S2. Molecular and clinical data by family

Family	Individual	Sex	cDNA	Protein	Clinical details (by family)
AGS102	P1	M	c.2159G>A	p.Arg720Gln	Pre-natal onset; congenital infection-like presentation (low platelets; hepatosplenomegaly; abnormal neurology). Severe DD and SD. Hypertension of undefined cause. Died age 2 years
AGS163	P1	M	c.2336G>A	p.Arg779His	Presented < 1 month of age with irritability, feeding difficulties, axial hypotonia and abnormal limb posturing. At age 13 years he demonstrated a severe quadriplegia, with no head control, no speech, abnormal ocular movements and microcephaly
AGS237 (LD_0762)	P1	M	c.1009A>G	p.Arg337Gly	Neuro-regression with spasticity and dystonia after normal development to age 15 months. At age 12 years he had no useful hand function, could not sit independently and had limited words, although his understanding was relatively preserved. Died age 16 years of cardiopulmonary arrest secondary to pulmonary hypertension
AGS259	P1	M	c.2336G>A	p.Arg779His	Apparently normal neonatal history but at age 6 months demonstrated axial hypotonia and lower limb spasticity. At 8 years of age he was severely delayed, never having acquired the ability to sit, with minimal hand function, gastrostomy feeding and no effective communication. Now, at 13 years of age he demonstrates an increasingly severe dystonic movement disorder. Father and paternal grandmother without disease features at 54 and 84 years of age respectively (the latter dying of coronary artery disease at that time)
	P2 (father of P1)	M			
	P3 (mother of P2)	F			

AGS376	P1	M	c.2335C>T	p.Arg779Cys	Likely prenatal onset with IUGR, poor feeding and developmental delay (obvious by age 4 months). At 29 months of age he developed a lupus-like illness with recurring fevers, weight loss, diarrhea, an ulcerative photosensitive vasculitic rash, hepatosplenomegaly, generalized lymphadenopathy, arthritis of the knees and ankles, serositis with pericardial effusion and a pronounced inflammatory response. Significant gastrointestinal vasculitis with rectal bleeds and liver dysfunction was a major problem and he died aged 3 years despite intensive immunosuppressive therapy
AGS524	P1	F	c.1483G>A	p.Gly495Arg	Delayed motor development with spasticity, then developing a transverse myelitis at age 21 months in association with a severe lupus-like illness and positive AQP4 antibodies. At age 10 years she exhibits increased tone with spasticity in all 4 limbs and clawing of the hands. She has good understanding, but her speech remains dysarthric, and she is considered to have a degree of intellectual delay. She has had surgery for scoliosis. Her father experienced the childhood-onset of lower-limb spasticity which has been very slowly progressive (to current age of 39 years). His upper limb function and intellect are fully preserved in the absence of any systemic features and with completely normal neuroimaging (brain and spine)
	P2 (father of P1)	M			
AGS531	P1	F	c.2544T>G	p.Asp848Glu	The proband developed walking difficulties with moderate spasticity of the lower limbs from 3 years of age, and at age 14 years was unable to walk on tip-toes. Cerebral CT scan demonstrated ICC. Her twin brother is asymptomatic, with unremarkable clinical examination and normal CT brain. The father of the proband experienced walking difficulties from 2 years of age, with a slowly progressive course. He could run when aged 24 years, but by 41 years of age he demonstrated severe spastic weakness with normal intellect. Brain CT at age 24 years revealed extensive ICC. The father's brother experienced progressive lower limb motor impairment since age 13 years. At 38 years of age, he demonstrated spastic weakness with normal cognition. Again, ICC was seen on CT. The proband's paternal grandfather was asymptomatic and without abnormal clinical signs at age 65 years
	P2 (brother of	M			

	P1)				
	P3 (father of P1 and P2)	M			
	P4 (brother of P3)	M			
	P5 (father of P3 and P4)	M			
AGS626	P1	M	c.1178A>T	p.Asp393Val	Mild transaminitis noted at age 3 months and microvesicular steatosis seen on liver biopsy at 1 year of age. Transaminase values gradually normalized by age 4 years. After exhibiting normal motor development to age 13 months, he experienced a rapid loss of motor and intellectual skills over a period of 1 month resulting in severe spastic tetraparesis, axial hypotonia and a loss of speech. At age 17 years he demonstrated a spastic tetraplegia with mild dystonic movements in all 4 limbs, communication was limited to eye movements and he had no useful hand function
AGS647	P1	M	c.2159G>A	p.Arg720Gln	Neonatal infection-like presentation (low platelets; anaemia). Developmental delay obvious by age 3 months with SD of the limbs and axial hypotonia. Concentric hypertrophy of the left cardiac ventricle diagnosed age 9 months, and acute nephrotic syndrome with focal glomerular sclerosis and tubuloreticular inclusions on renal biopsy at 10 months of age. Hypothyroidism identified at age 18 months and atrophic gastritis at 2 years of age. At this time he was severely developmentally delayed with poor head control, no useful hand function and limited social interaction
AGS674	P1	M	c.992C>G	p.Thr331Arg	Normal development to age 12 months when he developed a spastic tetraparesis with dystonia, dysarthria, mild intellectual deficit and a mixed sensory and motor neuropathy. White matter lesions and calcification on neuroimaging. He also exhibited growth failure, dental abnormalities with absence of many permanent teeth, and psoriatic-like skin lesions with hypochromic lentigines

AGS723	P1	F	c.2335C>T	p.Arg779Cys	Severe psoriatic-like lesions noted in the first months of life, with a progressive spastic paraparesis becoming evident during childhood and preserved intellect. Striatal calcification present on cerebral CT scanning
AGS735	P1	M	c.2471G>A	p.Arg824Lys	Normal development until age 12 months when he experienced psychomotor regression, with abnormal white matter on cerebral MRI. At 13 years of age he exhibited a spastic dystonic tetraparesis with normal head circumference. Verbal expressive language was absent but non-verbal intelligence quotient was within the normal range
AGS755	P1	M	c.1465G>A	p.Ala489Thr	Proband presented with chilblain-like lesions of the ears and erythematous cheeks at age 1 year. His father had exhibited similar ear lesions at 8 months of age and subsequently developed psoriatic-like lesions, lentigines, deformities of the hands and feet with tendon retractions and loss of secondary dentition after adolescence. The younger brother to the proband demonstrated early chilblain-like lesions and multiple lentigines, with delayed motor development and then a period of motor regression at age 2.5 years so that he was unable to walk or stand, having only a few spoken words at this age. Glaucoma identified soon thereafter
	P2 (brother of P1)	M			
	P3 (father of P1 and P2)	M			
AGS848	P1	M	c.1165G>A	p.Gly389Arg	The proband demonstrated a phenotype characteristic of AGS presenting in early infancy. His father exhibited a lower limb spasticity without other features. The father's maternal grandmother, his mother being deceased with unknown history, was reported to be asymptomatic at age 84 years (but was not formally examined)
	P2 (father of P1)	M			
	P3 (maternal grandmother of P2)	F			

AGS1001	P1	M	c.1347C>G	p.Asn449Lys	Development considered normal until age 18 months when concerns were raised about the need for continued support whilst walking. Over the following year he developed progressive spasticity in his lower limbs, and he demonstrated speech delay - not speaking until age 4 years. He developed autoimmune hypothyroidism at age 6 years. ICC identified at age 11 years. At age 18 years he was able to walk with two sticks, exhibited a marked startle response and had mild learning difficulties. He was normocephalic, had lower limb spasticity with normal upper limb examination, Raynaud phenomenon and psoriasis
AGS1004	P1	F	c.2335C>T	p.Arg779Cys	Perinatal thrombocytopenia, followed by pneumocystis carinii and CMV positive pneumonia requiring ventilation at age 3 months. Development considered normal until age 8 months when he lost skills and developed a SD, with widespread hyperintense white matter lesions on T2 MRI. Now, no motor skills, no speech and minimal comprehension, spastic dystonic tetraparesis, microcephaly, short stature, severe visual impairment, psoriatic-like lesions, glaucoma and nystagmus
AGS1156	P1	M	c.2335C>T	p.Arg779Cys	Normal development to age 8 months at which time he experienced a period of regression. The following 8 months were characterised by sterile pyrexias, irritability, increasing spasticity and exaggerated startle responses to sounds. At 16 months of age he was microcephalic, small, and centrally hypotonic with peripheral spasticity. As reported in Kothur et al. he has shown an apparently positive response to treatment with ruxolitinib, so that at age 5 years, having taken ruxolitinib 5mg bd for 3.5 years, he is making developmental gains, although he continues to demonstrate a spastic-dystonic tetraparesis. He crawls at speed independently, and has good functional use of his hands with finger feeding, and drives a motorised wheelchair with skill and good visuo-spatial accuracy. He uses his iPad to communicate, and can make requests and jokes. His repeat MRI shows no atrophy and cardiac examination remains normal
AGS1290	P1	M	c.2486C>G	p.Thr829Ser	The older sibling demonstrated apparently normal development in the first 5 months of life and then experienced rapid neurological decline, whilst his brother was always delayed. Both children are fully dependent for all activities, gastrostomy fed and exhibit a severe SD. Abnormal white matter and calcification seen on brain imaging, with a raised level of neopterin in the CSF. The proband developed seizures at age 7 years

	P2 (brother of P1)	M			
AGS1351	P1	F	c.2336G>A	p.Arg779His	Severe, early onset developmental delay with peripheral spasticity and truncal hypotonia. Died age 2 years
AGS1430	P1	M	c.2866A>G	p.Ile956Val	The proband experienced an episode of encephalitis at the age of 6 years, before which time his development was completely normal. He subsequently exhibited a progressive spastic paraparesis with abnormal white matter and cerebral calcification. His father experienced a similar onset of disease at age 2 years. Both individuals are of normal intellect with no other features. The putative mutation was inherited from the clinically asymptomatic paternal grandfather of the proband
	P2 (father of P1)	M			
	P3 (father of P2)	M			
AGS1504 (LD_1175)	P1	F	c.2159G>A	p.Arg720Gln	Early development possibly normal but after 1st DPT vaccination aged 2 months she experienced fevers with prolonged crying and change in feeding pattern. Persistent elevation of TSH noted at 3 months of age requiring long-term thyroxine replacement. Persistent feeding difficulties and failure to thrive led to gastrostomy placement at 16 months of age. Head control achieved by age 7 months, and sitting with support at 13 months of age. However, at age 16 months she developed fevers and experienced developmental regression with loss of skills. Now, she demonstrates profound intellectual disability with SD, remains hypothyroid, has a persistent thrombocytopenia and has undergone orthopaedic surgery for contractures and hip dislocations. She is under gynaecological review for premature adrenarche, and is small (height -4.7 SD; weight -2.46 SD) and microcephalic (OFC -4.5 SD). Progressive neurodegeneration with abnormal white matter and calcification on brain imaging
AGS1509	P1	M	c.2336G>A	p.Arg779His	Global developmental delay obvious by age 9 months, progressing to spastic quadriplegia with axial hypotonia and distal hypertonia. CSF pterins elevated. Cranial CT and brain MRI at 2 years of age showed basal ganglia and frontal / parietal lobe calcification with delayed myelination

AGS1514	P1	M	c.2465G>A	p.Arg822Gln	Developmental delay clear by age 6 months. At 12 months of age he was able to pull and stand, but these milestones were then lost and he subsequently developed spasticity with contractures of the knee joints and elbows. Brain MRI at age 2 years was normal, but calcification was seen on cerebral CT at 6 years of age. A chronic transaminitis was noted
AGS1938	P1	F	c.992C>T	p.Thr331Ile	All three affected family members exhibited a phenotype consistent with a diagnosis of SMS, variably demonstrating a deforming non-destructive arthropathy, abnormal dentition, aortic valve stenosis and psoriatic like lesions with multiple lentigenes
	P2 (mother of P1)	F			
	P3 (sister of P2)	F			
AGS1972	P1	F	c.992C>G	p.Thr331Arg	Father and daughter with a phenotype consistent with a diagnosis of SMS, variably demonstrating a deforming non-destructive arthropathy, abnormal dentition, psoriatic like lesions and glaucoma
	P2 (father of P1)	M			
AGS2081	P1	M	c.2561T>A	p.Met854Lys	Normal milestones until age 14 months when he developed a progressive spastic paraparesis, losing the ability to walk by age 4 years. Demyelinating sensory polyneuropathy diagnosed at 3 years of age. Wide-spread erythema and hyperkeratotic lesions on elbows and knees from an early age, with delayed tooth eruption and then precocious loss of permanent teeth. Glaucoma diagnosed at age 5 years. Brain MRIs demonstrating non-specific hypersignal of periventricular white matter initially, with dense and wide-spread calcification noted at age 9 years. Cognitive and language skills normal at 12 years of age

AGS2154_1 (LD_1240)	P1	M	c.2335C>T	p.Arg779Cys	No past medical history and normal development until onset of headache, dizziness and repeated falls at age 8 years. Brain MRI remarkable for extensive T2 high signal of white matter limited to the right hemisphere. Brain biopsy of right frontal cortex showed vasculopathy of unknown aetiology and myelin loss. Headaches have worsened, often associated with limb pain. Motor development, intellect and neurological examination considered normal. His mother, aged 38 years, demonstrates no clinical features (with normal interferon signature on 3 occasions)
AGS2154_2 (LD_1240.1)	P2 (mother of P1)	F			
AGS2177	P1	M	c.2336G>A	p.Arg779His	Psychomotor development was normal until age 12 months, at which time he had started to walk and babble. There then followed a sudden regression with loss of skills and the onset of a progressive spastic tetraparesis. At age 29 years he is spastic and wheelchair bound without useful hand function or recognisable words. The same mutation was identified in the proband's healthy sister and mother (aged 32 and 67 years respectively)
	P2 (mother of P1)	F			
	P3 (sister of P1)	F			
AGS2180	P1	F	c.2335C>T	p.Arg779Cys	Likely never normal development; head control at age 3 months, sitting at 9 months of age but never cruising, and there were significant parental concerns before her first birthday. Now, age 4 years, she demonstrates a significant SD of all four limbs, with no speech but good communication skills
AGS2222	P1	M	c.2471G>A	p.Arg824Lys	Isolated liver disease (neonatal hepatitis, bile duct paucity, liver fibrosis). Normal neurology; Now 7 years of age

AGS2369	P1	M	c.1747A>G	p.Ile583Val	Presented with delayed walking and expressive language. Found to have white matter abnormalities on brain MRI. Now, aged 9 years, has some truncal hypotonia and peripheral spasticity. He is wheelchair bound but is considered bright, using sign language and a communication aid
AGS2399	P1	M	c.2317G>C	p.Glu773Gln	Normal development until age 16 months, at which time he was walking. He then demonstrated psychomotor regression, with his condition stabilising by age 20 months. He is now left with a spastic diplegia and no expressive language
AGS2422	P1	F	c.2159G>A	p.Arg720Gln	Bilateral glaucoma at age 3 years. At 4 years of age she experienced walking difficulties and demonstrated a pyramidal syndrome and demyelinating neuropathy. Two episodes of neurological deterioration at age 17 years (post-trauma) and 22 years (post-partum), so that she became wheelchair bound. She demonstrates livedo reticularis, psoriasis, xerosis and chilblains. On brain MRI there are features of an occipital pseudo-stroke or vasculitis, associated with calcification of the basal ganglia. Vision has deteriorated over time with neovascularisation and cataract. She demonstrates a mild neutropenia and renal impairment. Head circumference is reduced (52 cm), but her intellect is normal
AGS2507	P1	F	c.2335C>T	p.Arg779Cys	This girl was microcephalic at birth, and then experienced a sudden onset of irritability and crying at three weeks of age, subsequently demonstrating severe psychomotor retardation (no eye contact or other communication), spastic tetraparesis and dystonia. Mild liver and spleen enlargement with elevated liver transaminases noted soon after presentation were still present at 10 months of age. MRI of the brain at age 6 months showed hypomyelination and calcifications
AGS2548	P1	M	c.2159G>A	p.Arg720Gln	Born at term weighing 1.67 kg (-3.76 SD) with features of sepsis and autoimmune thrombocytopenia. Developmental delay obvious by age 3 months, with white matter disease and intracranial calcification. Now at age 3 years he demonstrates severe psychomotor delay and a spastic dystonic tetraparesis

AGS2586	P1	M	c.1178A>C	p.Asp393Ala	Born at 38 weeks gestation in good condition but symmetrically small. Fed poorly and presented with fever at age 6 weeks. Anaemic, with thrombocytopenia, leucocytosis and hepatosplenomegaly which resolved although he had a persistent mild hepatic transaminitis. He was delayed in his development but made steady progress so that at age 18 months he had reduced truncal tone but could commando crawl, use pincer grasp bilaterally and had many purposeful words. At 21 months of age he began to regress, becoming irritable, losing the ability to commando crawl or lift his head when prone, becoming stiff, and developing hand fisting. He remained visually bright, and understood language but lost his expressed words. Cerebral MRI showed mild delayed myelination only, and CSF was acellular but with a raised neopterin of 307 nmol/l (normal <30)
AGS2662 (LD_1640)	P1	F	c.2404A>G	p.Asn802Asp	Development considered normal until 11 months of age when she became irritable and febrile, associated with a loss of skills. She had not achieved walking, but lost the ability to pull herself up and sit unassisted, and began to exhibit features of a SD with apparently normal cognition
AGS2669	P1	M	c.1331A>G	p.Glu444Gly	Presented in early infancy with severe irritability and developmental delay. MRI showed symmetrical white matter abnormalities, and intracranial calcification was observed on CT. He was very irritable and dystonic - needing to be carried much of the time. Pulmonary insufficiency became significant and he died at age 6 months
Hm_1	P1	F	c.2156C>T	p.Ala719Val	Neonatal presentation with growth retardation, microcephaly, abnormal neurology, anaemia and low platelets. She experienced a CMV pneumonia at age 3 months. At the age of 7 months she experienced further neurological deterioration coinciding with immunisation. Liver biopsy undertaken because of a persistent transaminitis suggested an autoimmune hepatitis (mild chronic hepatitis with mild interface activity, mild portal and perisinusoidal fibrosis) treated with steroids. Now, at age 27 months, she is severely developmentally impaired with no head control, a spastic tetraparesis and truncal hypotonia, no words, no reactive smiling and increased blood pressure

Berg_1	P1	F	c.2336G>A	p.Arg779His	Development normal until age 9 months when she became irritable and developed a progressive encephalopathy with abnormal white matter on cerebral MRI. At the age of 20 months a neuroblastoma was detected, leading to a diagnosis of opsoclonus-myoclonus syndrome in the absence of opsoclonus. By this time she demonstrated severe psychomotor retardation with no speech and a SD. At age 6 years she developed a lupus-like illness with skin rash and vasculitic lesions, raised ESR and normal CRP. Autoantibody titres were elevated and skin biopsy demonstrated findings compatible with SLE
Orc_0098	P1	M	c.2336G>A	p.Arg779His	Clinical onset at 8 months with irritability, disturbed sleep-wake pattern and fevers and frank psychomotor regression from 11 months with gradual appearance of microcephaly, spastic-dystonic tetraplegia. Abnormal white matter and calcification on cerebral imaging
LD_0940.0	P1	M	c.2342G>A	p.Gly781Glu	Mild developmental delay with frank regression at age 15 months, losing the ability to hold a bottle, sit and roll. He developed a diffuse panniculitis at this time. At 17 months of age cerebral MRI and CT documented intracranial calcification and leukoencephalopathy. Now he has a spastic-dystonic tetraparesis, is able to smile, vocalise and recognize familiar faces
LD_0943.0	P1	F	c.2342G>A	p.Gly781Glu	Presentation in the second year of life with toe-walking and lower limb spasticity which has been progressive. Brain Imaging showed calcification and leukodystrophy. Current neurologic evaluation demonstrates spastic diplegia with apparently intact cognition
LD_0982.0	P1	M	c.2159G>A	p.Arg720Gln	Neonatal thrombocytopenia and hepatosplenomegaly with failure to thrive. Persistent thrombocytopenia until 2 years of age despite IVIG and steroids. At age 9 months he was not sitting. Liver biopsy at 1 year of age was complicated by haemorrhage and associated with a loss of skills and the ability to feed. Cerebral MRI and CT demonstrated severe atrophy and leukodystrophy. Pulmonary hypertension diagnosed prior to his fourth birthday in the setting of acute illness, and confirmed by cardiac catheterization at the age of 7 years. Now demonstrates developmental delay with spastic quadriparesis and microcephaly

LD_1030.0	P1	F	c.2335C>T	p.Arg779Cys	Seen at age 4 months with developmental delay, petechiae, leucocytosis, thrombocytopenia, hepatosplenomegaly and elevated LFTs. Liver biopsy consistent with autoimmune hepatitis. Brain MRI at age 10 months revealed delayed myelination and diffuse calcifications. Now demonstrates developmental delay with spastic quadriparesis and microcephaly
LD_1067.0	P1	M	c.2336G>T	p.Arg779Leu	Developmental delay obvious by age 5 months. At age 1 year he could hold his head, babble and could use his hands to hold objects and feed himself with a spoon. Brain MRI showed some areas of high signal white matter on T2 sequences. At age 24 months he could use a walker with good trunk control. Repeat imaging at this time showed new white matter signal abnormalities and basal ganglia calcification. After a viral infection he experienced psychomotor regression, losing the ability to walk, sit with support and hand use, with evolving spastic dystonic tetraparesis. He now uses an eye gaze device for augmentative communication, a head switch for wheelchair control and has a gastrostomy
LD_1199.0	P1	F	c.2336G>A	p.Arg779His	Delayed motor milestones obvious by age 6 months. She learned to crawl, pull to stand, cruise and use up to 10 words. At 18 months of age, after a viral infection with a high fever, she experienced rapid neurological regression, developing lower limb spastic diplegia. She now crawls fisted, though she can still scribble and use a fork or spoon, with expressive speech and language delay. White matter T2 high signal on brain MRI at 2 years of age
LD_1346.0	P1	M	c.2936T>G	p.Leu979Trp	Respiratory distress with cardiomegaly and pulmonary hypertension recognised shortly after birth requiring intubation. He also demonstrated severe anaemia and thrombocytopenia requiring multiple transfusions. Brain imaging revealed calcifications and CSF neopterin was elevated. Gastrostomy tube placed at 2 months of age for poor feeding and growth. He died of respiratory problems at age 4 months

LD_1381.0 A (Hart)	P1	F	c.2336G>A	p.Arg779His	Development normal until 30 months when experienced regression with loss of walking in the absence of any cognitive deficit. Her younger brother demonstrated spasticity and mild motor deficit noted in first year of life, with apparently normal cognition despite abnormal white matter and calcification on brain imaging. The father of these two children was diagnosed with cerebral palsy as an infant. Toe walking and mild, spastic gait with basal ganglia calcification but otherwise well with no cognitive deficit. The paternal grandfather of the proband was clinically asymptomatic at age 68 years
LD_1381.0 B (Hart)	P2 (brother of P1)	M			
LD_1381.2 (Hart)	P3 (father of P1 and P2)	M			
LD_1381.3 (Hart)	P4 (father of P3)	M			
LD_1488.0	P1	F	c.2407A>T	p.Ile803Phe	Irritability and poor feeding noted by 6 weeks of age, with obviously delayed development by age 4 months. She acquired a social smile at 2 months of age, but later milestone have never been acquired. Neurologic exam is remarkable for truncal hypotonia and a SD
LD_1585.0	P1	F	c.2336G>A	p.Arg779His	Early history unavailable but increased leg tone noted by age 1 year treated with baclofen. She was able to cruise around furniture at age 20 months. Global developmental delay clear at 3 years of age. Currently has spastic tetraplegia

IFIH1 mutation annotation based on the reference cDNA sequence NM_022168.2

AGS: Aicardi-Goutières syndrome; CLL: Chilblain-like lesions; CMV: Cytomegalovirus; CRP: C-reactive protein; CSF: Cerebrospinal fluid; CT: Computed tomography; DD: Developmental delay; DPT: Diphtheria, pertussis, tetanus; ESR: Erythrocyte sedimentation rate; F: Female; LLD: Lupus-like disease; ICC: Intracranial calcification; IUGR: Intrauterine growth retardation; IVIG: Intravenous immunoglobulins; LFTs: Liver

function tests; M: Male; MRI: Magnetic resonance imaging; SD: Spastic dystonia; SLE: Systemic lupus erythematosus; SMS: Singleton-Merten syndrome; SP: Spastic paraparesis; TM: Transverse myelitis; TSH: Thyroid stimulating hormone

Supp. Table S3. Summary data of interferon stimulated gene expression testing in the Crow laboratory

Family	Individual	ISG scores (age)
AGS102	P1	Not available
AGS163	P1	Not available
AGS237 (LD_0762)	P1	12.26 (12.55); 22.70 (12.60)
AGS259	P1	19.22 (8.14); 35.92 (8.21); 26.72 (8.29)
	P2 (father of P1)	10.83 (48.80); 17.00 (48.87); 29.08 (48.95); 20.35 (49.73)
	P3 (mother of P2)	12.28 (79.33); 7.02 (79.41); 19.38 (80.19)
AGS376	P1	Not available
AGS524	P1	20.08 (3.91); 25.45 (5.19); 41.01 (5.83); 13.14 (7.36)
	P2 (father of P1)	15.17 (32.34); 11.51 (32.85); 17.85 (33.13); 29.34 (33.62); 9.90 (33.95); 15.99 (36.11); 22.33 (36.33); 24.39 (36.61)
AGS531	P1	53.82 (13.33); 9.51 (13.65)
	P2 (brother of P1)	17.59 (13.33); 13.46 (13.65)
	P3 (father of P1 and P2)	24.83 (39.87); 30.52 (40.15)
	P4 (brother of P3)	9.89 (36.97); 8.66 (37.24)
	P5 (father of P3 and P4)	9.16 (65.65); 6.23 (65.93)
AGS626	P1	12.36 (17.14); 15.63 (17.66)
AGS647	P1	22.03 (1.39); 16.12 (1.96); 18.41 (2.06)
AGS674	P1	21.77 (9.44)
AGS723	P1	39.45 (15.20); 16.08 (18.91)
AGS735	P1	18.86 (14.57); 20.10 (14.75)
AGS755	P1	7.54 (2.16); 21.33 (2.47); 59.77 (3.04)
	P2 (brother of P1)	7.29 (3.82); 31.92 (4.70)
	P3 (father of P1 and P2)	9.84 (41.00); 16.72 (41.88)
AGS848	P1	20.84 (3.52); 112.49 (4.30); 16.67 (6.80)
	P2 (father of P1)	23.7 (37.88); 11.95 (38.44); 5.90 (38.66); 9.86 (39.63); 17.54 (41.16)
	P3 (maternal grandmother of	11.96 (78.67); 11.14 (78.89); 10.08 (81.39)

	P2)	
AGS1001	P1	24.47 (14.72); 13.61 (17.46)
AGS1004	P1	20.21 (4.97)
AGS1156	P1	12.54 (1.66); 12.78 (1.81);12.78 (2.03); 10.22 (2.11); 5.08 (2.18); 8.71 (2.31)
AGS1290	P1	17.81 (3.79)
	P2 (brother of P1)	25.49 (5.30)
AGS1351	P1	12.49 (1.47); 10.43 (1.81)
AGS1430	P1	15.28 (10.60); 13.66 (10.67); 16.43 (10.86); 13.74 (11.29)
	P2 (father of P1)	19.82 (46.72); 24.55 (46.91); 16.24 (47.33)
	P3 (father of P2)	0.96 (69.10); 1.15 (69.52); 1.17 (69.66)
AGS1504 (LD_1175)	P1	Not available
AGS1509	P1	10.06 (5.06)
AGS1514	P1	7.23 (6.32)
AGS1938	P1	29.72 (18.31)
	P2 (mother of P1)	15.14 (45.35)
	P3 (sister of P2)	16.89 (27.34)
AGS1972	P1	14.03 (9.65)
	P2 (father of P1)	18.82 (47.53)
AGS2081	P1	15.38 (10.34)
AGS2154_1 (LD_1240)	P1	5.27 (10.56); 0.41 (10.65); 0.56 (10.75)
AGS2154_2 (LD_1240.1)	P2 (mother of P1)	0.53 (38.89); 0.36 (38.98); 0.34 (39.08)
AGS2177	P1	13.14 (29.97)
	P2 (mother of P1)	7.22 (62.69)
	P3 (sister of P1)	11.52 (33.19)
AGS2180	P1	12.11 (2.68); 23.14 (3.51); 14.94 (3.70); 5.62 (3.79); 9.85 (3.87); 13.30 (4.02); 7.13 (4.19)
AGS2222	P1	3.65 (5.26); 4.81 (5.51); 10.151 (7.14)

AGS2369	P1	16.49 (7.76); 12.32 (8.27)
AGS2399	P1	Not available
AGS2422	P1	18.03 (38.08)
AGS2507	P1	8.10 (0.82)
AGS2548	P1	Not available
AGS2586	P1	15.36 (3.04)
AGS2662 (LD_1640)	P1	Not available
AGS2669	P1	17.1 (0.42)
AGS2685	P1	Not available

Boxes coloured blue represent clinically asymptomatic individuals. Boxes coloured pale green define individuals with normal interferon scores (<2.466) on at least one occasion

Supp. Table S4. Details of previously published cases with *IFIH1* mutations that were not ascertained as part of the present series

cDNA change	Protein change	Report(s)	Associated phenotypes	Documented upregulation of interferon signalling	gnomAD frequency	Seen in the present study
c.1114C>T	p.Leu372Phe	Oda et al. (de novo)	AGS	Yes	Novel	No
c.1354G>A	p.Ala452Thr	Oda et al. (de novo)	AGS	Yes	Novel	No
c.2336G>A	p.Arg779His	Oda et al. (de novo); Van Eyck et al. (de novo)	AGS; SP-LLD	Yes	1/244230	Yes
c.2335C>T	p.Arg779Cys	Marguet et al. (de novo)	AGS	No	Novel	Yes
c.2439A>T	p.Glu813Asp	Amari et al. (de novo)	AGS	No	Novel	No
c.2465G>A	p.Arg822Gln	Rutsch et al. (2 familial; 1 de novo); Pettersson et al. (1 familial)	SMS	Yes	6/244096	Yes
c.2561T>A	p.Met854Lys	Takeichi et al. (de novo)	AGS-SMS	No	Novel	Yes

IFIH1 mutation annotation based on the reference cDNA sequence NM_022168.2

AGS: Aicardi-Goutières syndrome; LLD: Lupus-like disease; Spastic paraparesis; SMS: Singleton-Merten syndrome

* Note that we consider it unlikely that the c.961G>T / p.Glu321* variant described by Al Mutairi et al (Pediatr Neurol 2018;78:35-40) is responsible for the putative Aicardi-Goutières syndrome phenotype reported in that paper, given that all other such variants are gain-of-function due to missense substitutions, and noting that *IFIH1* is not highly constrained (i.e. multiple nonsense variants are recorded on gnomAD).

Amari, S., Tsukamoto, K., Ishiguro, A., Yanagi, K., Kaname, T., & Ito, Y. (2019). An extremely severe case of Aicardi-Goutieres syndrome 7 with a novel variant in IFIH1. *Eur J Med Genet*. doi:10.1016/j.ejmg.2019.04.003

Marguet, F., Laquerriere, A., Goldenberg, A., Guerrot, A. M., Quenez, O., Flahaut, P., . . . Nicolas, G. (2016). Clinical and pathologic features of Aicardi-Goutieres syndrome due to an IFIH1 mutation: A pediatric case report. *Am J Med Genet A, 170A*(5), 1317-1324.

Oda, H., Nakagawa, K., Abe, J., Awaya, T., Funabiki, M., Hijikata, A., . . . Heike, T. (2014). Aicardi-Goutieres syndrome is caused by IFIH1 mutations. *Am J Hum Genet, 95*(1), 121-125.

Pettersson, M., Bergendal, B., Norderyd, J., Nilsson, D., Anderlid, B. M., Nordgren, A., & Lindstrand, A. (2017). Further evidence for specific IFIH1 mutation as a cause of Singleton-Merten syndrome with phenotypic heterogeneity. *Am J Med Genet A, 173*(5), 1396-1399.

Rutsch, F., MacDougall, M., Lu, C., Buers, I., Mamaeva, O., Nitschke, Y., . . . Hennekam, R. C. (2015). A Specific IFIH1 Gain-of-Function Mutation Causes Singleton-Merten Syndrome. *Am J Hum Genet, 96*(2), 275-282.

Takeichi, T., Katayama, C., Tanaka, T., Okuno, Y., Murakami, N., Kono, M., . . . Akiyama, M. (2018). A novel IFIH1 mutation in the pincer domain underlies the clinical features of both Aicardi-Goutieres and Singleton-Merten syndromes in a single patient. *Br J Dermatol, 178*(2), e111-e113.

Van Eyck, L., De Somer, L., Pombal, D., Bornschein, S., Frans, G., Humblet-Baron, S., . . . Liston, A. (2015). IFIH1 mutation causes systemic lupus erythematosus with selective IgA-deficiency. *Arthritis Rheumatol, 67*(6), 1592-1597.
